# Retrospective evaluation of a robust hybrid planning technique established for irradiation of breast cancer patients with included mammary internal lymph nodes

**DOI:** 10.1186/s13014-022-02039-w

**Published:** 2022-04-15

**Authors:** Marina Hennet, Stephan Radonic, Uwe Schneider, Matthias Hartmann

**Affiliations:** 1 Department of Radiotherapy, Klinik Hirslanden AG, Rain 34, 5000 Aarau, Switzerland; 2 Department of Physics, University of Zürich, Zürich, Switzerland; 3 Division of Radiation Oncology-Small Animal Department, Vetsuisse Faculty, University of Zurich, Zürich, Switzerland

**Keywords:** Radiotherapy, Treatment planning, Hybrid, Breast cancer, Mammary interna, VMAT, IMRT, Optimization

## Abstract

**Background:**

The irradiation of breast cancer patients with included internal mammary lymph nodes challenges radiation planning with regard to robustness and protection of OARs. In this publication, a feasible hybrid radiation technique is presented with a retrospective dosimetric and radiobiological analysis of patient data of our institute from 2016 to 2020 and robustness analysis.

**Methods:**

The proposed hybrid irradiation technique consists of two IMRT tangents and two partial VMAT fields. The retrospective dosimetric and radiobiological evaluation are made for 217 patient treatments (right- and left-sided). The robustness is evaluated regarding an artificial swelling from 0.4 to 1.5 cm for a random example patient and compared to a pure VMAT planning technique with use of a virtual bolus. The out of field stray dose is calculated for a selected patient plan and compared to alternative radiation techniques.

**Results:**

The coverage D_95%_ of the PTV_Eval_ (with breast swelling of 1.5 cm) changes for the hybrid plan from 96.1 to 92.1% of prescribed dose and for the pure VMAT plan from 94.3 to 87%. The retrospective dosimetric evaluation of patient irradiations reveals a D_mean_ for total lung 6.5 ± 0.9 Gy (NTCP[Semenenko 2008] 2.8 ± 0.5%), ipsilateral lung 10.9 ± 1.5 Gy, contralateral lung 2.2 ± 0.6 Gy, heart 2.1 ± 1.1 Gy (ERR[Schneider 2017] 0.02 ± 0.17%) and contralateral breast 1.7 ± 0.6 Gy. The scatter dose of the hybrid irradiation technique is higher than for pure VMAT and lower than for pure IMRT irradiation.

**Conclusions:**

The feasibility of the proposed planning technique is shown by treating many patients with this technique at our radiotherapy department. The hybrid radiation technique shows a good sparing of the OARs in the retrospective analysis and is robust with regards to a breast swelling of up to 1.5 cm. The slightly higher stray dose of the hybrid technique compared to a pure VMAT technique originates from higher number of MUs and lower conformity.

**Supplementary Information:**

The online version contains supplementary material available at 10.1186/s13014-022-02039-w.

## Background

Radiotherapy is an important part of the treatment of breast cancer, along with surgery and chemotherapy. Radiotherapy is mostly used adjuvant to reduce the likelihood of loco regional recurrence and improve patient survival [1, 2]. An EORTC study performed in 2015 [3] shows evidence that the irradiation of the internal mammary lymph nodes is beneficial for some of the patients in terms of disease-free survival, distant disease-free survival and breast-cancer mortality. If breast or chest wall irradiations includes the internal mammary lymph nodes (MI LN), the irradiation with a conventional 3 dimensional conformal technique (3DCRT) while simultaneously fulfilling all planning constraints is very challenging [4]. In general, a large portion of the lung must be included in the irradiation field and the coverage of the supra clavicular lymph nodes (supra) as well as the MI LN is not optimal. Several studies [5–8] indicate, that a good sparing of the organs at risk (OARs), target coverage and conformity may be achieved by a pure volumetric modulated arc (VMAT) or hybrid planning technique where arcs and static intensity modulated (IMRT) fields are combined. Therefore, our clinic developed a hybrid planning technique that allows the necessary sparing of the OARs, target coverage and robustness with respect to swelling of the breast tissue during therapy. This hybrid planning technique consists of two IMRT tangents serving mainly the breast region and two VMATs irradiating the supra as well as the MI LN. Most of our breast cancer patients with included MI LN are irradiated with this hybrid planning technique since 2016. In this publication, the robustness of the hybrid planning technique is examined. The dosimetric data of all patients irradiated with the hybrid technique are evaluated from 2016 to 2020. The normal tissue complication probabilities for selected organs are calculated and additionally, the out of field organ stray doses are compared with other planning techniques.

## Methods

### Patient selection and delineation of target volume and organs at risk

217 patient treatments with the hybrid planning technique are evaluated regarding their relevant dosimetric parameters. These patients are treated between January 2016 and December 2020 at the Radiotherapy Department of the Hirslanden AG in Zürich, Männedorf and Aarau in Switzerland. Three planning target volumes (PTVs) are contoured separately. Firstly the $\text {PTV}_{\mathrm{Breast}}$ is defined as the whole breast tissue or breast wall of the affected side of the patient with a 3–5 mm cropping towards the skin. The second volume $\text {PTV}_{\mathrm{Supra}}$ encompasses parts of level 2 as well as Level 3 and 4 of the axilla, whereas the third volume $\text {PTV}_{\mathrm{MI}}$ encloses the MI LN.

The dose prescription is done for the PTVs to either 50 Gy (2 Gy/fract, 5 fract/week) or 42.4 Gy (2.65 Gy/fract, 5 fract/week). For some $\text {PTV}_{\mathrm{Supra}}$ and $\text {PTV}_{\mathrm{MI}}$ the prescription dose is 90% of the prescribed dose to the $\text {PTV}_{\mathrm{Breast}}$. Those volumes are excluded from the evaluation. The plans are normalized such that the PTVs get a mean dose of $100\pm 2$% of the prescribed dose while the dose-volume constraint $\text {V}_{95\%} > 95\%$ should be fulfilled for PTVs. The patients are positioned head first supine on a breast board with the arms in an overhead position. All patients are irradiated in deep inspiration breath hold with the real time Position Management respiratory gating technique (Varian Medical Systems) to reduce dose interference effects from intra fractional motion, to reduce the volume of irradiated lung tissue and to gain distance towards the heart for left sided breast cancer treatments. Included are both patients with right and left sided breast cancer. To get an evaluation of a rather homogenous patient cohort, treatments with use of a physical bolus are not included in the dosimetric evaluation. The inclusion of treatments with a physical bolus would have enlarged the standard deviation of the PTV coverage. The irradiation of a boost volume is not included in the dosimetric evaluation.

The following OARs are contoured:

- Lungs
  The lungs are contoured separately as ipsilateral and contralateral lung and together yielding the total lung.
- Heart
  The whole heart is contoured, starting inferiorly of the left pulmonary artery down to the diaphragm. The fatty tissue of the pericardium is included in the structure, as the cardiac vessels run there.
- Spinal cord
  The spinal cord is delineated at least along the whole length of the $\text {PTV}_{\mathrm{Supra}}$.
- Esophagus
  The esophagus is delineated on the levels where dose from the VMAT fields may be expected.
- Contralateral breast
  The contralateral breast is contoured to contain the whole breast tissue of the contralateral side of the patient.

### Hybrid planning technique

All patients are planned using a planning CT with a dedicated treatment planning system (TPS) Eclipse (Varian Medical Systems, Version 13.6 and 15.6). The hybrid technique consists of two tangents covering the $\text {PTV}_{\mathrm{Breast}}$, such as two arcs used for the coverage of the $\text {PTV}_{\mathrm{MI}}$ and $\text {PTV}_{\mathrm{Supra}}$.

The isocenter is set longitudinally at the boarder of breast and supra. The ventral and lateral position of the isocenter are chosen to be as close as possible to the $\text {PTV}_{\mathrm{MI}}$ so that the VMAT field for MI LN coverage is minimized, resulting in smooth leaf motions of the multi leaf collimator (MLC). First two opposing IMRT tangents are setup excluding the MI LN. The collimator angle is chosen such that one jaw spares as much volume of the lung and heart as possible while simultaneously the field provides still coverage of the $\text {PTV}_{\mathrm{Breast}}$ excluding the $\text {PTV}_{\mathrm{MI}}$. The two tangents are named as the lateral tangent and the medial tangent. The intersections of the tangents towards the $\text {PTV}_{\mathrm{MI}}$ and $\text {PTV}_{\mathrm{Supra}}$ are manually smoothed such that the transition between arcs and static IMRT fields is fluent. The optimal fluence of the medial tangent is reduced by 30% over the length of the $\text {PTV}_{\mathrm{MI}}$. In the study of Balaji et al. [9] the proportion of 70–80% 3DCRT and 20–30% VMAT is found to be optimal for a hybrid VMAT technique which is close to the splitting of 85% dose from the tangents and 15% from VMAT to the $\text {PTV}_{\mathrm{Breast}}$ in this study.

**Fig. 1:**
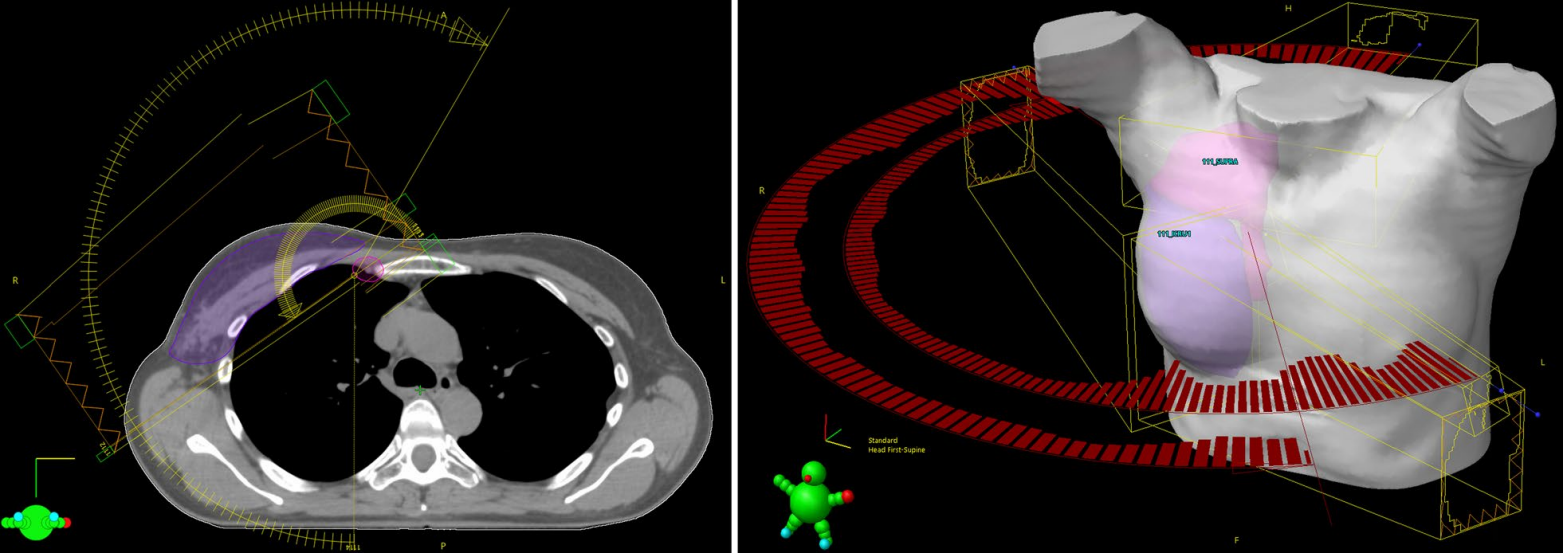
The Axial and 3D model view of the beam setup of the hybrid planning techniques consisting of two IMRT tangents and two VMATs

In a second step, two coplanar arcs (VMAT) are setup on the same isocenter as the tangents. One arc is taking care of the $\text {PTV}_{\mathrm{Supra}}$ coverage running approximately from 0°to 180°, while the second arc coveres the $\text {PTV}_{\mathrm{MI}}$ running from the angle of the medial to the angle of the lateral tangent. The beam setup is shown in Fig. 1.

The photon optimization algorithm PO (Varian Medical Systems, Version 13.6 and 15.6) is used for inverse optimization of the plans. The plans are calculated with the Anisotropic Analytical Algorithm (AAA) of Eclipse (Varian Medical Systems, Version 13.6 and 15.6).

The plans are iteratively optimized over several steps using either the tangents as baseplan for the VMAT optimization or vice versa. The clinical goals that should be fulfilled in the inverse planning process are chosen based on the Swiss Group for Clinical Cancer Research (SAKK) 23/16-Taxis phase 3 trial study protocol [10]. The planners are advised to further limit the OAR dose, while the PTV coverage and homogeneity are not compromised.

### Robustness analysis

In the case of a breast irradiation, a possible breast swelling should already be compensated during treatment planning. This compensation is done with a skin flash, when using the common technique of two opposing tangents. A skin flash is done by expanding the fluence map outside the body outline with a nearest cell fill method, such that the $\text {PTV}_{\mathrm{Breast}}$ is fully covered even when a swelling of the breast occurs. With the described hybrid planning technique the $\text {PTV}_{\mathrm{Breast}}$ receives at least 85% of its dose from the tangents having a skin flash. The missing 15% dose of the $\text {PTV}_{\mathrm{Breast}}$ is delivered by the VMAT field dedicated to deliver the dose to the $\text {PTV}_{\mathrm{MI}}$.

The described technique is compared to a pure VMAT technique with two arcs covering the breast region and MI LN and one arc covering the supra-clavicular region. The optimization of the pure VMAT technique is done on the planning CT extended with a virtual bolus of 1 cm thickness with − 500 HU [11] in the breast region, to open the leafs of the arcs towards the skin for breast swelling compensation. The optimized plan is then applied to the original planning-CT and the monitor units (MUs) of the arcs are scaled without additional optimization of the MLC movement [12].

The robustness of the planning technique with regard to PTV coverage and influence on OAR dose is analysed by planning a randomly chosen right-sided breast cancer patient. A plan using the described hybrid technique and a pure VMAT plan are setup. To simulate different levels of breast swelling, the body contour is expanded in the breast region by 0.4 cm, 0.7 cm, 1.0 cm and 1.5 cm with $HU=0$ [13]. The two patient plans are applied to the extended body contour and the dose distributions recalculated with fixed MU values from the plan without breast swelling. For the evaluation, a structure $\text {PTV}_{\mathrm{Eval}}$ is generated as expanded $\text {PTV}_{\mathrm{Breast}}$ towards the swelling with 4 mm skin cropping.

### Dosimetric and radiobiological parameters

Various dosimetric parameters are retrospectively evaluated from 217 patient hybrid treatment plans. The evaluated parameters may be found in Table 1. In addition to dose-volume parameters, some geometric and normal tissue complication probabilities (NTCP) are evaluated. As the prescription dose varies within the patient cohort, the absolute dosimetric parameters are additionally evaluated with renormalized dose volume histograms (DVHs) to a fractionation of $25\times 2$ Gy. Therefore, the interpretation of the data is more straight forward. The NTCP values are only calculated for the DVHs with the original fractionation, to remain at an estimation of risk for the actual patient treatments.

**Table 1:** All dose-volume, biological and geometrical parameters which are evaluated retrospectively for the hybrid patient treatments

Structure	Dose-volume parameter	Biological parameter	Geometric parameter
PTVs	D_min_ (%), D_mean_ (%), D_max_ (%), D_2%_ (%), D_50%_ (%), D_95%_ (%), D_98%_ (%), V_90%_ (%), V_95%_ (%)		volume (cm^3^), length (cm)
Lung ipsilateral	D_mean_ (Gy), V_20Gy_ (%)	NTCP (%) (Semenenko 2008) n = 1, m = 0.35, TD_50_ = 37.6Gy, $\alpha /\beta$ = 3	Volume (cm^3^)
Lung contralateral	D_mean_ (Gy), V_5Gy_ (%)		Volume (cm^3^)
Lung total	D_mean_ (Gy), V_20Gy_ (%)	NTCP (%) (Semenenko 2008) n = 1, m = 0.41, TD_50_ = 29.9Gy, $\alpha /\beta$ = 3	Volume (cm^3^)
Heart	D_mean_ (Gy), V_5Gy_ (%), V_25Gy_ (%)	NTCP (%) (Schneider 2017) s = 0.75, $\gamma$ = 1.29, D50 = 36.5 Gy	Volume (cm^3^)
Breast contralateral	D_mean_ (Gy), V_5Gy_ (%)		Volume (cm^3^)
Spinal cord	D_max_ (Gy), D_2%_ (Gy)		Volume (cm^3^), length (cm)
Esophagus	D_mean_ (Gy), D_2%_ (Gy)	NTCP (%) (Belderbos 2005) n = 0.69, m = 0.36, TD_50_ = 47 Gy, $\alpha /\beta$ = 3	Volume (cm^3^), length (cm)

Regarding the lung toxicity, the NTCP-model of Semenenko et al. [14] with the endpoint of symptomatic radiation pneumonitis is used. The model is based on the Lyman–Kutcher–Burman model formalism [15]. The evaluation of the risk for acute esophagitis grade 2+ is done with the NTCP-model parameter of Belderbos et al. [16]. Additionally, the excess relative risk (ERR) for major coronary events is calculated using the relative seriality model with the model parameters of Schneider et al. [17].

### Stray doses for hybrid irradiation technique

The analytical model for stray dose calculation of Hauri et al. [18] is used to calculate the out of field dose distribution. The model is composed of three different components: a mechanical model for the patient scatter and empirical models for collimator scatter and head leakage, respectively and predicts the stray dose distribution with an average local difference of around 11%. The hybrid plan is set up on a randomly chosen sample patient and compared to a 3DCRT plan containing four static fields with dynamic wedges without intensity modulation, a static IMRT-only plan with five fields and a plan containing solely three VMAT fields. The prescribed fractionation is set to $25\times 2$ Gy = 50 Gy and the clinical goals for the inverse planning process are chosen based on the Swiss Group for Clinical Cancer Research (SAKK) 23/16-Taxis phase 3 trial study protocol [10]. Despite the large differences in the various planning techniques, the goal is to meet our clinical specifications while maintaining equivalent dosimetric plan quality.

For the whole body dose calculation a virtual phantom from the National Cancer Institute (NCI) is used [18]. It represents a woman with same height and weight as the actual sample patient. All important organs and tissues are contoured. The evaluation of the stray dose is done on the structure set of the Whole-Body-Dose representation phantom. The match of the virtual phantom and the example patient is done on the structure of the right lung.

The 3DCRT is composed of four static 6MV fields with in total 480 MU. The pure IMRT plan uses five static 6MV IMRT fields with a total number of MUs of 1173. Two static IMRT fields with two VMAT fields with 6MV and 972 MU are used in the case of the hybrid planning technique and the pure VMAT plan is composed of three VMAT fields with in total 882 MU. The patient plans in the evaluation of our hybrid technique take an average of 940.9 ± 113.1 MU (217 plans). Therefore, the hybrid example plan is representative for the evaluated patient cohort.

## Results

### Robustness analysis

The resulting DVHs of the robustness analysis of our hybrid technique and the pure VMAT technique can be found in the Additional file 1: : A.1.

**Fig. 2:**
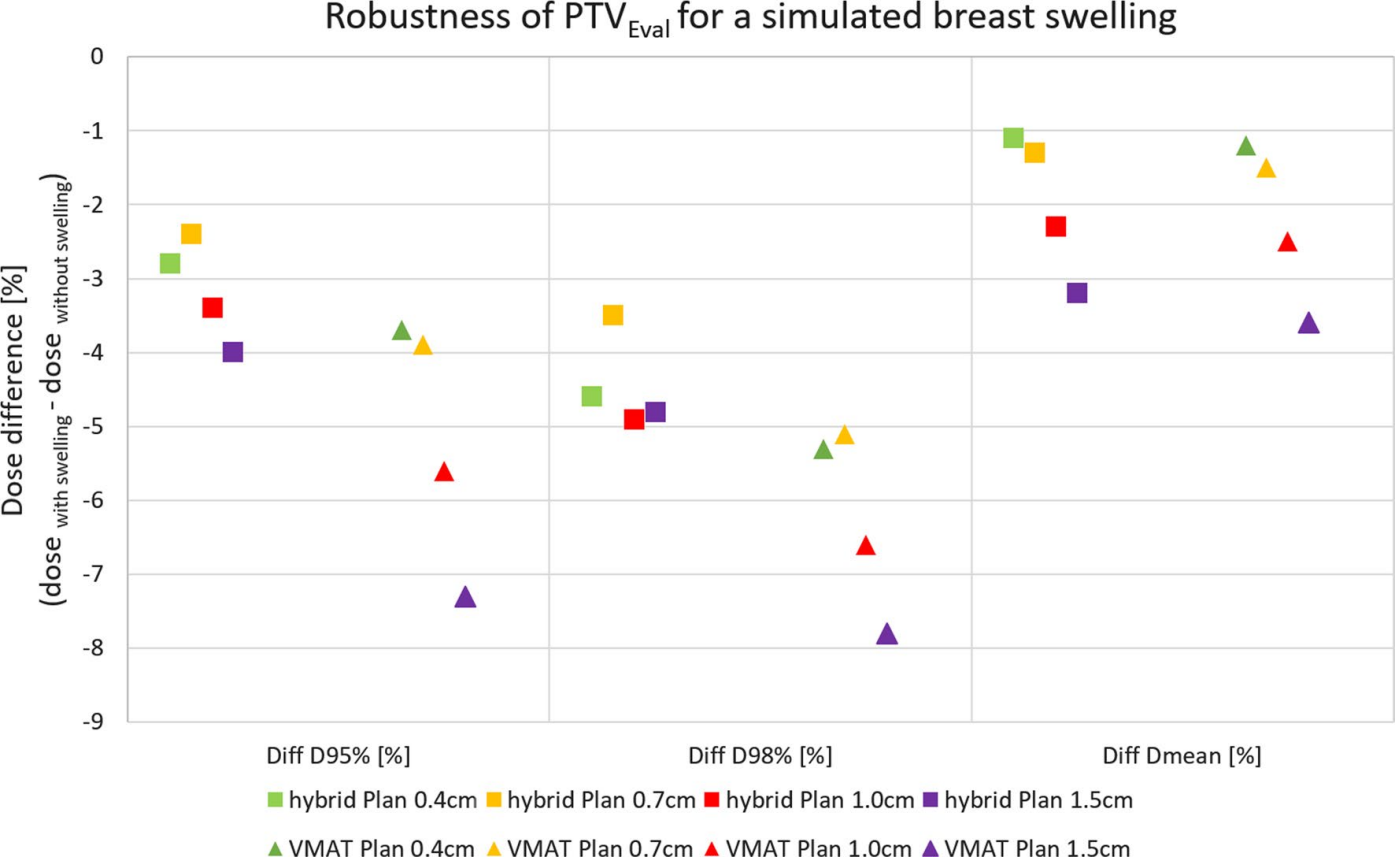
The dose difference (%) of D_95%_, D_98%_ and mean dose of the $\text {PTV}_{\mathrm{Eval}}$ of the original plan without breast swelling and the plan with a simulated breast swelling of 0.4 cm, 0.7 cm, 1.0 cm and 1.5 cm for the two planning techniques: hybrid (rectangles) and pure VMAT planning (triangles)

The coverage of the $\text {PTV}_{\mathrm{Breast}}$ is slightly decreased with the simulated swelling for both planning techniques compared to the original plan without swelling of the breast. The mean dose of the $\text {PTV}_{\mathrm{Eval}}$, which represents the complete $\text {PTV}_{\mathrm{Breast}}$ including swelling, changes from 100.3% (0 cm swelling) to 97.1% (1.5 cm swelling) and D_95%_ from 96.1 to 92.1% of prescribed dose for the hybrid and from 100 to 96.4% and from 94.3 to 87%, respectively for the pure VMAT plan. The mean dose of the $\text {PTV}_{\mathrm{Supra + MI}}$ changes from 100.2 to 100.4% of prescribed dose for both planning techniques. No significant change in the dose distribution within the OARs could be found. In Fig. 2 the comparison of the two planning techniques is illustrated by the dose differences relative to the plan without swelling for different evaluation dose-volume points.

### Dosimetric and radiobiological evaluation of patient treatments

The retrospective evaluation of the patient treatments with the hybrid technique is done for the PTVs for relative dose regarding the patient individual prescribed dose. The D_95%_ for the $\text {PTV}_{\mathrm{Breast}}$, $\text {PTV}_{\mathrm{Supra}}$ and $\text {PTV}_{\mathrm{MI}}$ with the original fractionation are 94.2 ± 3.7% , 95.4 ± 1.7% and 94.9 ± 2.7% respectively. The doses are specified as mean dose ± one standard deviation.

For the original fractionation, the D_mean_ of the total lung results in 6.5 ± 0.9 Gy, for the contralateral lung in 2.2 ± 0.6 Gy and the $\text {V}_{\mathrm{20Gy}}$ of the ipsilateral lung in 18.9 ± 3.7 Gy. The NTCP values regarding symptomatic pneumonitis are just insignificantly lower for the model of the ipsilateral lung with 2.1 ± 0.6% compared with result of the total lung model of 2.8 ± 0.5%. The mean dose to the heart is 2.1 ± 1.1 Gy and the relative seriality model results in an ERR of 0.02 ± 0.17% for major coronary events. Statistics are also evaluated separately for all left (103 patients) and all right (114 patients) breast irradiations, particularly to asses cardiac dose. The mean heart dose for the left-sided breast cancer patients D_mean_ = 2.3 ± 1.1 Gy does not vary significantly to the right-sided patients D_mean_ = 2.0 ± 1.1 Gy, such as the NTCP values show no significant difference: NTCP (left-sided) = 0.05 ± 0.25% versus NTCP (right-sided) = 0 ± 0.01%. The maximum dose to the spinal cord is 17.1 ± 4.4 Gy. The esophagus receives a mean dose of 10.5 ± 3.4 Gy and a calculated NTCP of 1.9 ± 1.2% for acute esophagitis grade 2+. The mean dose to the contralateral breast is 1.7 ± 0.6 Gy. In Table 2 all results of the evaluation for the single structures PTVs and OARs can be found for all patients together: left-sided and right sided. The separated evaluation regarding the heart dose is added as Additional file 1:  A.2. Additionally the population DVHs are shown in the Additional file 1: A.3 as the median DVH of all matched structures and the maximum, upper quartile (75%), lower quartile (25%) and minimum of all single DVHs for PTVs and OARs. The upper quartile (75%) is defined as the DVH where 75% of all single DVH points are below and the lower quartile (25%) is defined as the DVH where 75% of all single DVH points are above. All shown population DVHs are generated from the individual relative DVHs of the patients, each of which is normalized to the prescribed dose of the particular patient.

**Table 2:** The statistical data [mean ± standard deviation] for all three PTVs and OARs are shown for selected dose-volume Points and NTCP values for the patient data from 2016 to 2020

Parameters	PTV_Breast_			PTV_Supra_			PTV_MI_	
	Original fractionation			Original fractionation			Original fractionation	
# matched volumes	217			135			170	
Volume (cm^3^)	664.3 ± 331			166.7 ± 121.8			22.3 ± 14.1	
Length cr–cd (cm)	16.3 ± 1.8			5.3 ± 2.4			9.7 ± 1.9	
D_mean_ (%)	100.5 ± 1			100.3 ± 0.9			100 ± 1.2	
D_50%_ (%)	101 ± 1			100.6 ± 0.9			100.3 ± 1.1	
D_max_ (%)	111.1 ± 2.2			108.9 ± 2			108 ± 2.1	
D_98%_ (%)	91.2 ± 6.4			93.6 ± 2.1			93.5 ± 3.2	
D_95%_ (%)	94.2 ± 3.7			95.4 ± 1.7			94.9 ± 2.7	
D_2%_ (%)	105.9 ± 1.5			105 ± 1.5			104.9 ± 1.7	
D_min_ (%)	57.9 ± 21.9			80.1 ± 7			85.3 ± 6.1	
V_90%_ (%)	98.6 ± 2			99.5 ± 0.8			99 ± 2.6	
V_95%_ (%)	94.2 ± 3.6			95.2 ± 3.6			93.2 ± 8.2	

	Lung total		Lung contralateral		Lung ipsilateral		Heart	
	Original fractionation	25 × 2Gy = 50 Gy	Original fractionation	25 × 2Gy = 50 Gy	Original fractionation	25 × 2Gy = 50 Gy	Original fractionation	25 × 2Gy = 50 Gy
# matched volumes	213		216		216		212	
Volume (cm^3^)	4468.5 ± 789.8		2213.9 ± 470.1		2237.2 ± 420.7		529.7 ± 103.5	
D_mean_ (Gy)	6.5 ± 0.9	6.6 ± 0.9	2.2 ± 0.6	2.2 ± 0.6	10.9 ± 1.5	11 ± 1.4	2.1 ± 1.1	2.2 ± 1.1
V_5Gy_ (%)			10.2 ± 5.9	10.7 ± 5.8			10.9 ± 9.7	11.3 ± 10.1
V_20Gy_ (%)	9.5 ± 1.9	9.6 ± 1.8			18.9 ± 3.7	19.2 ± 3.5		
V_25Gy_ (%)							0.1 ± 0.5	0.1 ± 0.5
NTCP (%)	2.8 ± 0.5				2.1 ± 0.6		0.02 ± 0.17	
Model:	Semenenko 2008: symptomatic pneumonitis total lung				Semenenko 2008: symptomatic pneumonitis ipsilateral lung		Schneider 2017: relative seriality model: long-term cardiac mortality	

	Spinal cord		Esophagus		Breast contralateral			
	original fractionation	25 × 2Gy = 50 Gy	original fractionation	25 × 2Gy = 50 Gy	original fractionation		25 × 2Gy = 50 Gy	
# matched volumes	214		139		84			
Volume (cm^3^)	35.9 ± 15.3		14.5 ± 6.4		789.1 ± 409.7			
Length cr–cd (cm)	18.9 ± 7.4		11.4 ± 4					
D_mean_ (Gy)	5.4 ± 2.6	5.5 ± 2.6	10.5 ± 3.4	10.7 ± 3.4	1.7 ± 0.6		1.7 ± 0.6	
D_max_ (Gy)	17.1 ± 4.4	17.4 ± 4.4						
D_2%_ (Gy)	14.7 ± 3.8	15 ± 3.8	20.9 ± 6.1	21.2 ± 6.1				
V_5Gy_ (%)					2.4 ± 3.9		2.5 ± 4	
NTCP (%)			1.9 ± 1.2					
Model			Belderbos 2005: acute esophagitis grade 2+					

The whole evaluation for the PTVs is done with the relative DVHs and for the OARs with the absolute DVHs. The evaluation for the OARs is done for the original fractionation schemes of the plans and additionally with renormalized DVHs for a uniform fractionation of $25\times 2$ Gy = 50 Gy

### Stray dose distribution for hybrid irradiation technique

All plans but the 3DCRT plan are clinically acceptable. The dosimetric plan quality of the 3DCRT is inferior to that of the other plans and confronts the patient with a very high dose in the ipsilateral lung and insufficient PTV coverage.

**Fig. 3:**
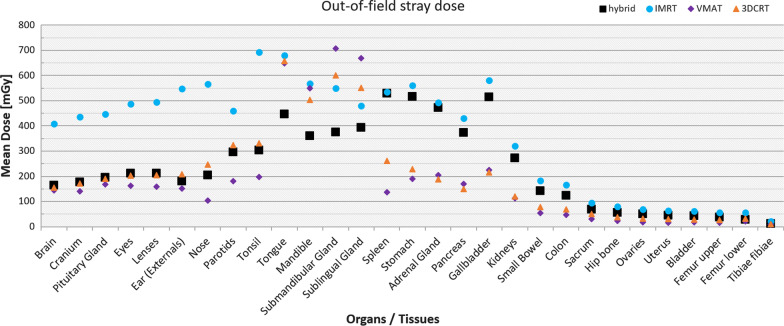
Comparison of mean organ doses (mGy) in the stray dose regime of the virtual phantom for a complete treatment with 50 Gy prescribed dose. The data of the hybrid plan is displayed in black squares, the pure IMRT plan in blue circles, the pure VMAT plan in violet diamonds and the 3D conformal plan in orange triangles

The four different planning techniques have the following ascending order with respect to stray dose: pure VMAT, 3DCRT, hybrid and pure IMRT. The comparison of the mean organ doses for various organs in the out of field region for a complete treatment with 50 Gy prescribed dose is shown in Fig. 3. A table of the absolute values can be found in the Additional file 1: A.4. The dose in the uterus may be seen as a representative of the stray dose for a fetus in a pregnant woman. The mean dose to the uterus is 45 mGy, 64 mGy, 30 mGy and 16 mGy for the hybrid, pure IMRT, 3DCRT and pure VMAT plan respectively.

## Discussion

### Robustness of the hybrid planning technique

The complexity in treatment planning is increased for a hybrid planning technique as well as for the pure VMAT planning with a virtual bolus compared to simply apply a skin flash to opposing tangents to minimize the effect of breast swelling. To obtain high quality plans, plans of both techniques must be generated by experienced planners.

Due to the skin flash of the tangents, the hybrid technique is robust with respect to swelling of the breast. The robustness is similar to a conventional breast irradiation with fluence modulated tangents, as solely 15% of the whole dose to the $\text {PTV}_{\mathrm{Breast}}$ is given with the VMAT technique. In the region of the supra-clavicular and internal mammary lymph nodes no swelling is expected. Therefore, robustness is given in this regime of the PTVs as well.

The pure VMAT technique shows a similar robustness as the described hybrid technique only up to a swelling of the breast of about 0.7 cm. This result is consistent with statements made in the publication by Rossi et al. [19]. Up to which extent of breast swelling the pure VMAT plan is robust, depends on the chosen HU and thickness of the virtual bolus in the previous planning process [11]. In order to obtain a robust plan with the VMAT technique, one makes use of the addition of the virtual bolus. However, this leads to the fact that the anatomy in the inverse optimization has to be changed and differs from the anatomy on which the final dose calculation is based on. In this point, an analogy can be drawn to the so-called convergence error, which occurs by the use of inaccurate dose calculation in inverse treatment planning in addition to the systematic error [20]. Hereby the convergence error results due to the optimization algorithm converges to a solution based on an inaccurate dose distribution, which is different from the optimal solution for the accurate dose calculation. A similar convergence error results for optimization based on the inaccurate patient anatomy. By artificially modifying the anatomy with the virtual bolus for optimization, the optimization of inverse treatment planning with VMAT converges to the solution based on an incorrect patient anatomy. Therefore, the choice of the thickness and HUs of the virtual bolus is not straightforward. A compromise must be found between minimal dosimetric impact and maximal robustness against breast swelling, which is investigated by Lizondo et al. [11]. Such kind of anatomical convergence error does not exist with the described hybrid planning technique. The robustness with respect to swelling of the breast is easily achieved by expanding the fluence of the IMRT tangents as much as needed with minimal dosimetric influence on the optimization and OAR doses. Therefore, it is possible to find a feasible compromise between sparing of the lung and other OARs and a robust plan which is insensitive to swelling of the breast. The breast swelling is only one aspect related to the robustness of a plan. On the other hand, inter- and intra fractional motion should be considered. The inter fractional motion can be kept as small as possible per example by using IGRT and the intra fractional motion by using DIBH.

Adaptive treatment planning is advancing, but it is not yet used as standard for breast irradiation. However, as an outlook the application is very interesting for indications like breast irradiation with included supra and MI LN, since the breast swelling must not be accounted for in advance any more, but the plan can be adjusted to the anatomy at the day of the treatment. This would eliminate the need for the planning-intensive Hybrid technique with its sequential VMAT and IMRT optimization steps or the use of a virtual bolus with pure VMAT planning. Therefore adaptive treatment planning may be listed as an additional possibility to cope with compensation of breast swelling beside the two planning techniques of our hybrid technique with skin flash and pure VMAT with virtual bolus. With adaptive treatment planning, the planer is again free to use any best possible field configuration. The disadvantage of sequential VMAT and IMRT optimization steps for hybrid technology could disappear by an optimizer that handles VMAT and IMRT fields simultaneously in one optimization step. Thus, it could also become an interesting field configuration for adaptive treatment planning.

### Dosimetric and radiobiological evaluation of patient treatments

The publication of Lin et al. [5] finds a superiority of the hybrid planning technique compared to pure arc planning and pure IMRT planning in regards of organ sparing. The retrospective dosimetric evaluation of the patient treatments in this study is in the same regime as the dosimetric results of Lin et al.

The evaluation of the $\text {PTV}_{\mathrm{Breast}}$ data has some pitfalls as the cropping of the $\text {PTV}_{\mathrm{Breast}}$ towards the skin ranged from 3 to 5 mm. Nevertheless, the PTV coverage for all three PTVs is satisfying our clinical requirements. The evaluation of the absolute dose parameters is done with the patient’s original fractionation and a second time with the renormalized DVH for a $25\times 2$ Gy fractionation.

In the publication of Doi et al. [8], a hybrid planning technique is compared to 3DCRT planning regarding OAR sparing, target coverage and homogeneity. Table 3 is based on the literature review regarding lung dose in the paper of Doi et al. and places the data of this study in context with the dataset [7–9, 21–24]. The table is extended with the corresponding data of the contralateral breast dose. The data of this study is in the same range as those of Doi et al. although in our study, the MI LN are included in the PTV. This is an indication that the hybrid planning technique is also robust in terms of plan quality regarding OAR sparing.

With the hybrid technique low mean lung doses can be achieved referring to Table 3. Since the hybrid technique delivers a large portion of the dose via the tangents, the contralateral lung receives only a small portion of the dose from the VMAT, which covers the PTV_MI_ while the ipsilateral lung stays within the clinical constraints. The lung NTCP-model of Semenenko for symptomatic pneumonitis concerning the DVH of the total lung provides an mean NTCP of 2.8 ± 0.5%. On the other hand, looking at Semenenko’s NTCP model, which only considers the DVH of the ipsilateral lung, it is questionable to use it. The more the planning deviates from pure tangents, the less accurate the model will be, since it was designed for pure tangent planning. The VMAT dose fraction arriving in the contralateral lung is not considered with this model.

**Table 3:** This comparison of the lungs and contralateral breast is recreated from the literature review in a publication of Doi et al. [8]

Publication	Ipsilateral lung		Contralateral lung		Total lung		Contralateral breast	
	V_20Gy_ (%)	D_mean_ (Gy)	V_5Gy_ (%)	D_mean_ (Gy)	V_20Gy_ (%)	D_mean_ (Gy)	V_5Gy_ (%)	D_mean_ (Gy)
Ma et al. [21] 6–12 field IMRT, MI LN incl.	28% ± 2	15.1 Gy ± 1.7	12% ± 11	2.3 Gy ± 1.3	–	–	2% ± 1	0.98 Gy ± 0.46
Nicols et al. [22] 2 field VMAT, MI LN incl.	23.3% ± 0.8	–	37.8% ± 4.9	–	16.3% ± 0.2	–	–	1.5 Gy ± 0.1
Zhao et al. [23] 2 field VMAT, MI LN incl.	17.7% ± 4.1	7.9 Gy ± 2.2	–	–	10.3% ± 5.7	6.5 Gy ± 16.6	–	-
Lai et al. [24] 2 field VMAT, MI LN excl.	23.1% ± 2.3	13.5 Gy ± 0.6	44.5% ± 6.5	5.1 Gy ± 0.7	–	–	–	3.1 Gy ± 0.3
Boman et al. [7] diff VMAT techniques, MI LN partly incl.	26–37%	14.4–18.6 Gy	3.0–2.7%	0.7–4.1Gy	–	–	6–40%	2–6 Gy
Balaji et al. [9] hybrid (3DCRT + VMAT), MI LN excl.	23–24%	12.7–14.3 Gy	0.07-11.9%	–	–	–	–	–
Doi et al. [8] hybrid (IMRT + VMAT), MI LN excl.	23.7% ± 6.4	12.0 Gy ± 2.4	5.2% ± 4.0	1.3 Gy ± 0.6	11.8% ± 3.3	6.7 Gy ± 3.8	–	–
This study hybrid (IMRT + VMAT), MI LN incl.	18.9% ± 3.7	10.9 Gy ± 1.5	10.2% ± 5.9	2.2 Gy ± 0.6	9.5% ± 1.9	6.5 Gy ± 0.9	2.4% ± 3.9	1.7 Gy ± 0.6

The evaluation of the data in this study is done with the original DVHs without renormalization

The optimal sparing of the heart has a high priority in our clinic. A mean heart dose of $2.1 \pm 1.1$ Gy is achieved. In this evaluation treatments of right- and left-sided breast cancer are evaluated together. With the ERR-calculation of the model of Schneider et al. [17] a result of $0.02 \pm 0.17$% ERR for major coronary events is achieved. It appears that the heart dose of right and left sided breast cancer patients in the hybrid technique is not significantly different. Since all patients are treated with DIBH no large dose contribution from the opposing tangents is expected. Probably the reason is, that most of the dose in the heart comes from the VMAT covering the MI LN. In the study of van der Bogaard et al. [25] the most prognostic dose-volume parameter to estimate acute coronary events is the volume of the left ventricle receiving 5 Gy ($\text {V}_{\mathrm{5Gy}}$). A hybrid technique is, regarding the heart dose, a mixture of the dose volume concept “a lot of dose to a little volume” (IMRT tangents) and “a little dose to a lot of volume” (VMAT). It would be very interesting to evaluate the dosimetric data of the left ventricle or left descending artery as suggested by Piroth et al. [26] and compare it with other planning techniques.

The results of the dosimetric evaluation of the spinal cord and the esophagus show a large scatter of the data. This fact can be caused by the relative geometric position to the PTVs, in particular by the relative position to the $\text {PTV}_{\mathrm{Supra}}$ which is dependent on the extent of the $\text {PTV}_{\mathrm{supra}}$ due to the patient’s medical history and anatomy.

The mean dose to the contralateral breast could be kept in mean below 2 Gy ($\text {D}_{\mathrm{mean}}: 1.7\pm 0.6$ Gy). The low mean dose is due to the fact, that the $\text {PTV}_{\mathrm{Breast}}$ is mainly covered by the tangents which contribute no direct irradiation to the contralateral breast. In the comparison with other studies in Table 3, the mean dose to the contralateral breast in of this study is in the lower range of the studies mentioned.

There are retrospective evaluations of irradiation of bilateral breast cancer patients with a pure VMAT technique [27]. We applied our hybrid technique to some bilateral breast cancer patients as well. Unfortunately, the amount of data is still too small for a meaningful statistical evaluation. As an outlook, it would be very interesting to compare the clinical data of our hybrid technique used for bilateral breast cancer patients with other techniques.

### Stray doses for hybrid irradiation technique

The evaluation of the out of field stray dose for the different planning techniques results in the lowest scatter dose for the pure VMAT plan and 3DCRT plan. Since the 3DCRT plan cannot meet the planning objectives, intensity modulation is needed, which is directly reflected in higher MUs and therefore a higher dose from collimator scatter and head leakage. The hybrid plan results in a higher amount of stray dose than the pure VMAT plan but less than the pure IMRT plan. The scattering radiation model takes into account phantom scatter, collimator scatter and head leakage. The phantom scatter is smaller for more conformal plans such as the pure VMAT plan or the hybrid plan, as stated by Ruben et al. [28]. The head leakage and collimator scatter depend mainly on the number of irradiated MUs. The sample hybrid plan to evaluate the stray dose uses 972 MU where as the evaluated patient treatments use in mean $940.9 \pm 113.1$ MU (217 plans). Therefore, the patient cohort is reasonably represented. The higher amount of stray dose of the hybrid plan compared to a pure VMAT plan is coming from higher number of irradiated MUs and lower conformity. Therefore, focus should be kept on minimizing the MUs in the optimization process and thus keeping the stray dose as small as possible. Regarding the advantages for conformity, coverage, OAR sparing and planning robustness, the amount of stray dose is no strict argument against a hybrid planning technique, but with respect to very young or pregnant patients this aspect should be considered.

## Conclusions

We conclude that the proposed hybrid planning technique is feasible in terms of robustness, applicability, planning capability and results in good OAR sparing. The sparing of OARs, especially the sparing of the ipsilateral lung is comparable to breast irradiations without inclusion of the MI LN. A good robustness of a plan with the presented hybrid technique is achievable by applying a skin flash on the tangents. The excellent sparing of OARs combined with the sparing of the contralateral lung and breast, the good target homogeneities and the robustness of the plan allow the conclusion that the proposed hybrid technique is at least equal if not superior to other comparable techniques. Therefore, the benefits outweigh the additional planing effort. The stray dose for the hybrid technique is higher than for the pure VMAT plan, which is a result of higher number of MUs of the hybrid plan and lower conformity.

## Supplementary Information

**Additional file 1.** This supplementary material provides additional information generated as part of the study. Section A.1 shows the DVHs of the robustness analysis of the hybrid and pure VMAT planning techniques. Section A.2 contains the dosimetric evaluation of the treatments with the hybrid technique, divided into left-sided and right-sided patient treatments with respect to cardiac dose. Section A.3 shows all population DVHs, boxplots and histograms of the dosimetric evaluation of the hybrid technique treatments of each evaluated structure. In section A.4, a table comparing the mean organ doses for four planning techniques (hybrid, pure IMRT, pure VMAT, and 3DCRT) is shown.

## Data Availability

The data that support the findings of this study are available from corresponding author [M. H.] but restrictions apply to the availability of these data, which were used under data protection laws for the current study, and so are not publicly available. Data are however available from the authors upon reasonable request and with permission of the patients.

## Abbreviations

MI LN: Internal mammary lymph nodes
3DCRT: Three dimensional conformal radiotherapy
supra: Supra clavicular lymph nodes
VMAT: Volumetric modulated arc
OAR: Organ at risk
IMRT: Intensity modulated radiotherapy
PTV: Planning target volume
TPS: Treatment planning system
MLC: Multi leaf collimator
MU: Monitor unit
AAA: Anisotropic Analytical Algorithm
NTCP: Normal tissue complication probability
DVH: Dose volume histogram
ERR: Excess relative risk

## References

[1] Group EBCTC, et al. Effect of radiotherapy after breast-conserving surgery on 10-year recurrence and 15-year breast cancer death: meta-analysis of individual patient data for 10 801 women in 17 randomised trials. Lancet 2011;378(9804):1707–16. 10.1016/S0140-6736(11)61629-2 PMC3254252 22019144

[2] McGale P, Correa C, Cutter D, Duane F, Ewertz M, Gray R, Mannu G, Peto R, Whelan T, Darby S (2014). Effect of radiotherapy after mastectomy and axillary surgery on 10-year recurrence and 20-year breast cancer mortality: meta-analysis of individual patient data for 8135 women in 22 randomised trials. Lancet.

[3] Poortmans PM, Collette S, Kirkove C, Van Limbergen E, Budach V, Struikmans H, Collette L, Fourquet A, Maingon P, Valli M (2015). Internal mammary and medial supraclavicular irradiation in breast cancer. N Engl J Med.

[4] Dogan N, Cuttino L, Lloyd R, Bump EA, Arthur DW (2007). Optimized dose coverage of regional lymph nodes in breast cancer: the role of intensity-modulated radiotherapy. Int J Radiat Oncol Biol Phys.

[5] Lin J-F, Yeh D-C, Yeh H-L, Chang C-F, Lin J-C (2015). Dosimetric comparison of hybrid volumetric-modulated arc therapy, volumetric-modulated arc therapy, and intensity-modulated radiation therapy for left-sided early breast cancer. Med Dosim.

[6] Zhang Q, Yu XL, Hu WG, Chen JY, Wang JZ, Ye JS, Guo XM (2015). Dosimetric comparison for volumetric modulated arc therapy and intensity-modulated radiotherapy on the left-sided chest wall and internal mammary nodes irradiation in treating post-mastectomy breast cancer. Radiol Oncol.

[7] Boman E, Rossi M, Haltamo M, Skyttä T, Kapanen M (2016). A new split arc vmat technique for lymph node positive breast cancer. Phys Med.

[8] Doi Y, Nakao M, Miura H, Ozawa S, Kenjo M, Nagata Y (2020). Hybrid volumetric-modulated arc therapy for postoperative breast cancer including regional lymph nodes: the advantage of dosimetric data and safety of toxicities. J Radiat Res.

[9] Balaji K, Yadav P, BalajiSubramanian S, Radha CA, Ramasubramanian V (2018). Hybrid volumetric modulated arc therapy for chest wall irradiation: for a good plan, get the right mixture. Phys Med.

[10] Henke G, Knauer M, Ribi K, Hayoz S, Gérard M-A, Ruhstaller T, Zwahlen DR, Muenst S, Ackerknecht M, Hawle H (2018). Tailored axillary surgery with or without axillary lymph node dissection followed by radiotherapy in patients with clinically node-positive breast cancer (taxis): study protocol for a multicenter, randomized phase-III trial. Trials.

[11] Lizondo M, Latorre-Musoll A, Espinosa N, Coral A, Cases C, Jornet N, Carrasco P, Delgado-Tapia P, Ruiz-Martinez A, Valverde-Pascual I (2019). Optimal parameters to perform the pseudo skin-flash on vmat on breast radiotherapy. Radiother Oncol.

[12] Tyran M, Tallet A, Resbeut M, Ferre M, Favrel V, Fau P, Moureau-Zabotto L, Darreon J, Gonzague L, Benkemouche A (2018). Safety and benefit of using a virtual bolus during treatment planning for breast cancer treated with arc therapy. J Appl Clin Med Phys.

[13] Seppälä J, Vuolukka K, Virén T, Heikkilä J, Honkanen JTJ, Pandey A, Al-Gburi A, Shah M, Sefa S, Koivumäki T (2019). Breast deformation during the course of radiotherapy: the need for an additional outer margin. Phys Med.

[14] Semenenko V, Li X (2008). Lyman–Kutcher–Burman NTCP model parameters for radiation pneumonitis and xerostomia based on combined analysis of published clinical data. Phys Med Biol.

[15] Allen Li X, Alber M, Deasy JO, Jackson A, Ken Jee K-W, Marks LB, Martel MK, Mayo C, Moiseenko V, Nahum AE (2012). The use and QA of biologically related models for treatment planning: short report of the TG-166 of the therapy physics committee of the AAPM. Med Phys.

[16] Belderbos J, Heemsbergen W, Hoogeman M, Pengel K, Rossi M, Lebesque J (2005). Acute esophageal toxicity in non-small cell lung cancer patients after high dose conformal radiotherapy. Radiother Oncol.

[17] Schneider U, Ernst M, Hartmann M (2017). The dose–response relationship for cardiovascular disease is not necessarily linear. Radiat Oncol.

[18] Hauri P, Hälg RA, Besserer J, Schneider U (2016). A general model for stray dose calculation of static and intensity-modulated photon radiation. Med Phys.

[19] Rossi M, Virén T, Heikkilä J, Seppälä J, Boman E (2021). The robustness of vmat radiotherapy for breast cancer with tissue deformations. Med Dosim.

[20] Jeraj R, Keall P, Schlegel W, Bortfeld T (2000). Errors in inverse treatment planning based on inaccurate dose calculation. The use of computers in radiation therapy.

[21] Ma J, Li J, Xie J, Chen J, Zhu C, Cai G, Zhang Z, Guo X, Chen J (2013). Post mastectomy linac IMRT irradiation of chest wall and regional nodes: dosimetry data and acute toxicities. Radiat Oncol.

[22] Nichols GP, Fontenot JD, Gibbons JP, Sanders ME (2014). Evaluation of volumetric modulated arc therapy for postmastectomy treatment. Radiat Oncol.

[23] Zhao L-R, Zhou Y-B, Sun J-G (2016). Comparison of plan optimization for single and dual volumetric-modulated arc therapy versus intensity-modulated radiation therapy during post-mastectomy regional irradiation. Oncol Lett.

[24] Lai Y, Chen Y, Wu S, Shi L, Fu L, Ha H, Lin Q (2016). Modified volumetric modulated arc therapy in left sided breast cancer after radical mastectomy with flattening filter free versus flattened beams. Medicine.

[25] van den Bogaard VA, Ta BD, van der Schaaf A, Bouma AB, Middag AM, Bantema-Joppe EJ, van Dijk LV, van Dijk-Peters FB, Marteijn LA, de Bock GH (2017). Validation and modification of a prediction model for acute cardiac events in patients with breast cancer treated with radiotherapy based on three-dimensional dose distributions to cardiac substructures. J Clin Oncol.

[26] Piroth MD, Baumann R, Budach W, Dunst J, Feyer P, Fietkau R, Haase W, Harms W, Hehr T, Krug D (2019). Heart toxicity from breast cancer radiotherapy. Strahlenther Onkol.

[27] Fiorentino A, Mazzola R, Naccarato S, Giaj-Levra N, Fersino S, Sicignano G, Tebano U, Ricchetti F, Ruggieri R, Alongi F (2017). Synchronous bilateral breast cancer irradiation: clinical and dosimetrical issues using volumetric modulated arc therapy and simultaneous integrated boost. Radiol Med (Torino).

[28] Ruben JD, Lancaster CM, Jones P, Smith RL (2011). A comparison of out-of-field dose and its constituent components for intensity-modulated radiation therapy versus conformal radiation therapy: implications for carcinogenesis. Int J Radiat Oncol Biol Phys.

