# Supplementary material for: Retrospective evaluation of a robust hybrid planning technique established for irradiation of breast cancer patients with included mammary internal lymph nodes
Source: Radiat Oncol. 2022 Apr 15;17:76. doi: 10.1186/s13014-022-02039-w (PMC9013158; doi:10.1186/s13014-022-02039-w)
Supplement: Supplementary file 1 — Additional file 1. This supplementary material provides additional information generated as part of the study. Section A.1 shows the DVHs of the robustness analysis of the hybrid and pure VMAT planning techniques. Section A.2 contains the dosimetric evaluation of the treatments with the hybrid technique, divided into left-sided and right-sided patient treatments with respect to cardiac dose. Section A.3 shows all population DVHs, boxplots and histograms of the dosimetric evaluation of the hybrid technique treatments of each evaluated structure. In section A.4, a table comparing the mean organ doses for four planning techniques (hybrid, pure IMRT, pure VMAT, and 3DCRT) is shown. [file 13014_2022_2039_MOESM1_ESM.pdf]

Supplemental material

A.1 DVHs of the Robustness Analysis

The resulting DVHs of the robustness analysis of our hybrid technique and the pure VMAT technique can be found in figure A1. The hybrid technique is illustrated with rectangles and the pure VMAT technique with triangles.

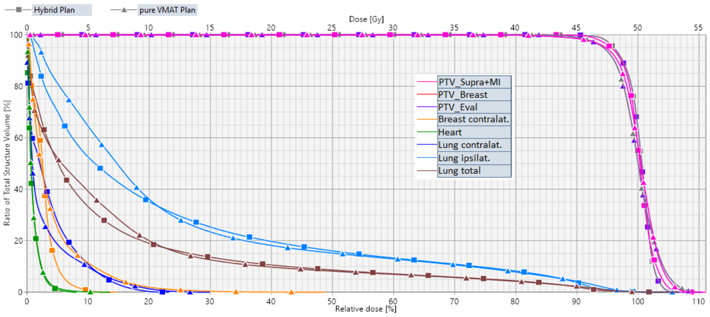

(a) No breast swelling

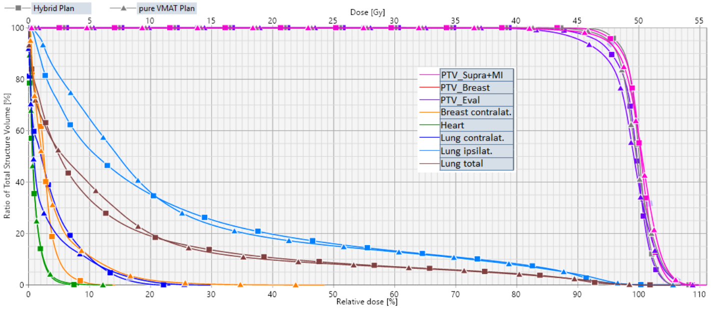

(b) 0.4cm breast swelling

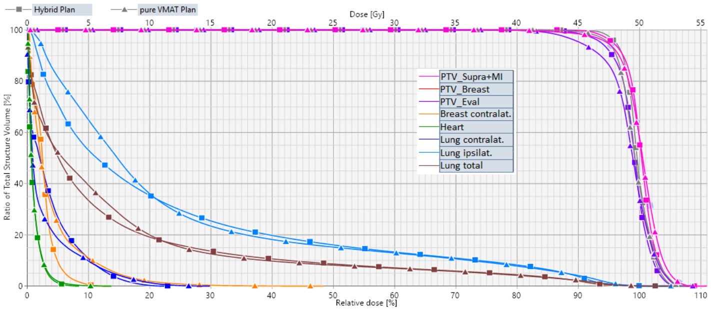

(c) 0.7cm breast swelling

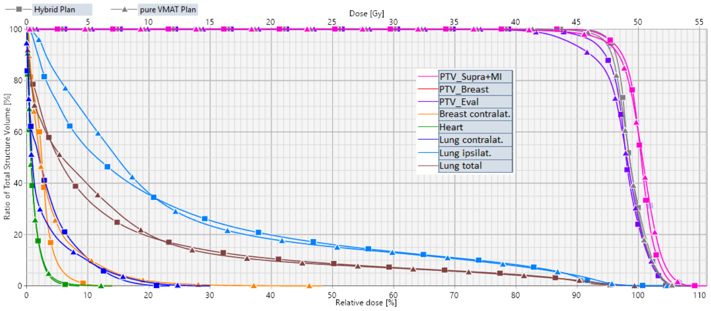

(d) 1.0cm breast swelling

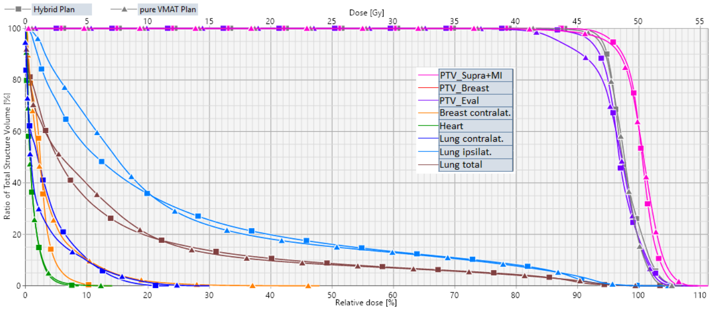

(e) 1.5cm breast swelling

Fig. A1: Comparison of a hybrid planed breast irradiation (rectangles) and pure VMAT (triangles) with included supra-clavicular and internal mammary lymph nodes for a simulated breast swelling of 0.4 cm to 1.5 cm.

A.2 Separated dosimetric evaluation of left-sided and right-sided patient treatments regarding heart dose

In figure A2 the data of the heart dose may be found separately for left- and right-sided irradiations. The evaluation was done for the original fractionation schemes of the plans and additionally with renormalized DVHs for a uniform fractionation of 25 x 2Gy = 50Gy.

	Heart left-sided patients		Heart right-sided patients	
	original	25 x 2Gy = 50Gy	original	25 x 2Gy = 50Gy
# matched Volumes	103		109	
Volume [cm^3]	528.4 ± 99.2		530.9 ± 107.8	
D_{mean} [Gy]	2.3 ± 1.1	2.3 ± 1.2	2 ± 1.1	2 ± 1.1
V_{5Gy} [%]	10.7 ± 9.7	11.1 ± 10.1	11.1 ± 9.6	11.6 ± 10.1
V_{20Gy} [%]				
V_{25Gy} [%]	0.2 ± 0.7	0.2 ± 0.7	0 ± 0	0 ± 0
NTCP [%]	0.05 ± 0.25		0 ± 0.01	
Model:	Schneider 2017: relative seriality model: long-term cardiac mortality		Schneider 2017: relative seriality model: long-term cardiac mortality	

Fig. A2: The statistics [mean±standard deviation] for the heart is shown for selected Dose-Volume Points for the patient data from 2016 to 2020 separated in left-sided and right-sided irradiations.

A.3 DVH of evaluation of all patient treatments

In the following figures and tables all results of the evaluation for the single structures may be found. A population DVH is shown as the median DVH of all matched structures and the maximum, upper quartile (75%), lower quartile (25%) and minimum of all single DVHs. The upper quartile (75%) is defined as the DVH where 75% of all single DVH points lay below and the lower quartile (25%) is defined as the DVH where 75% of all single DVH points are above. All shown Population-DVHs originate from relative DVHs normalized to the individual prescribed dose.

A.3.1 Additional information: PTV_{Breast}

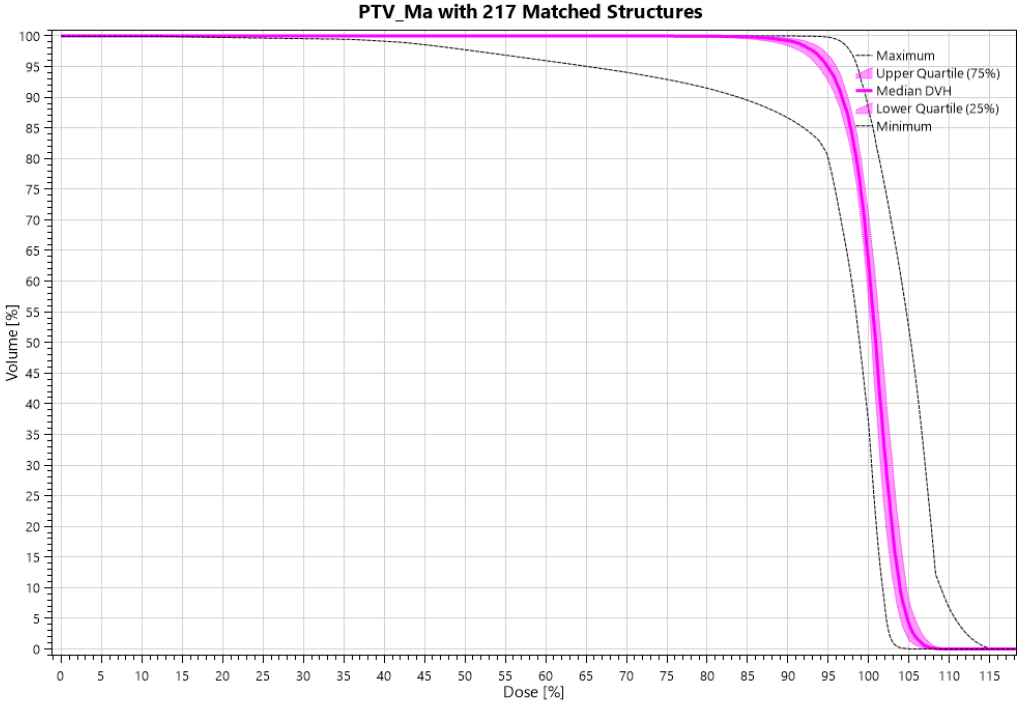

(a) Population DVH of PTV_{Breast}

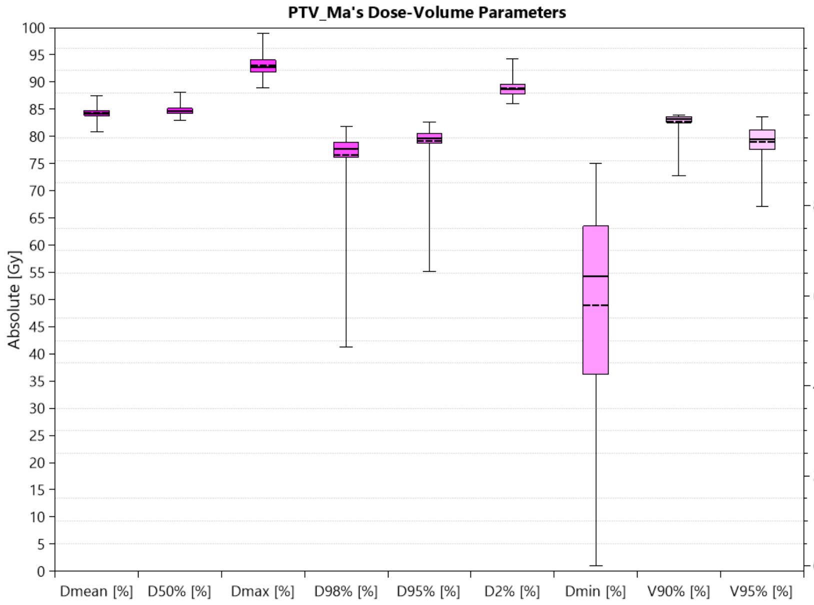

(b) Dose-Volume parameter of PTV_{Breast}

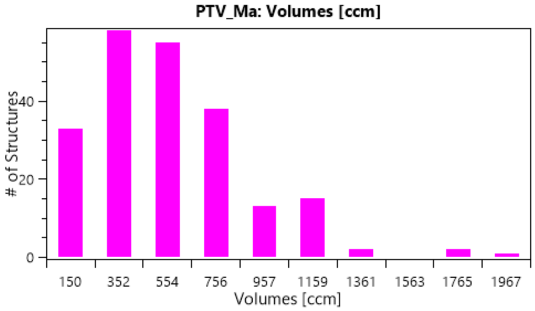

(c) Volumes of PTV_{Breast}

Fig. A3: (a) Median DVH of all matched structures PTV_{Breast} is shown. Additionally specified is the maximum, upper quartile (75%), lower quartile (25%) and minimum of all single DVHs. In (b) Boxplots of the dose-volume parameters and (c) histogram of the volume of all matched structures PTV_{Breast} is shown.

A.3.2 Additional information: PTV_{Supra}

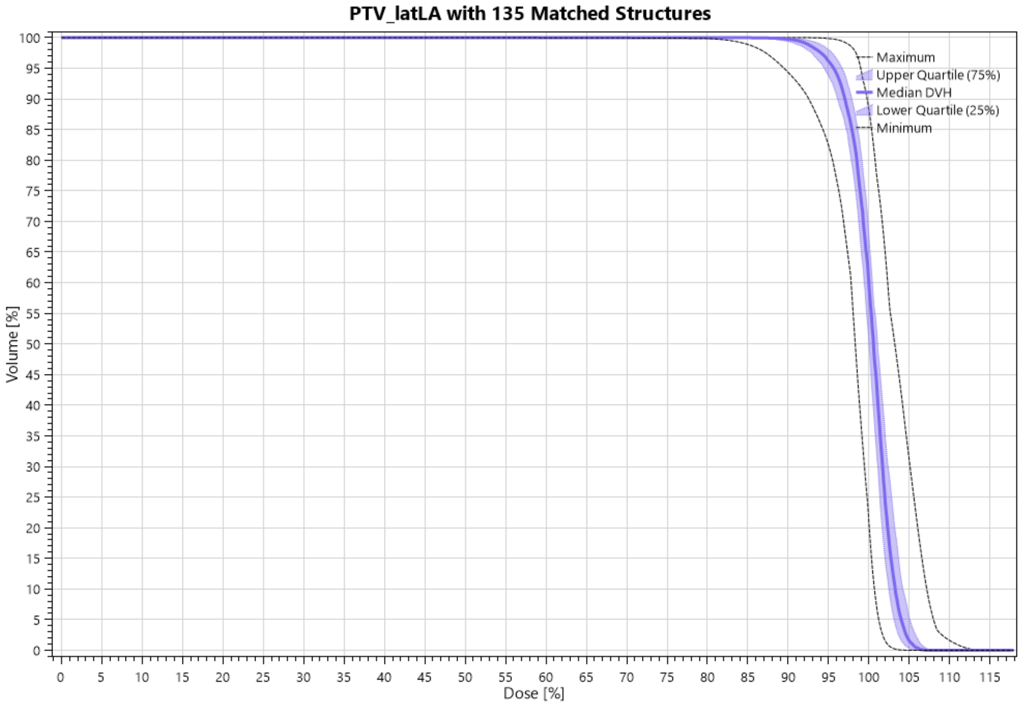

(a) Population DVH of PTV_{Supra}

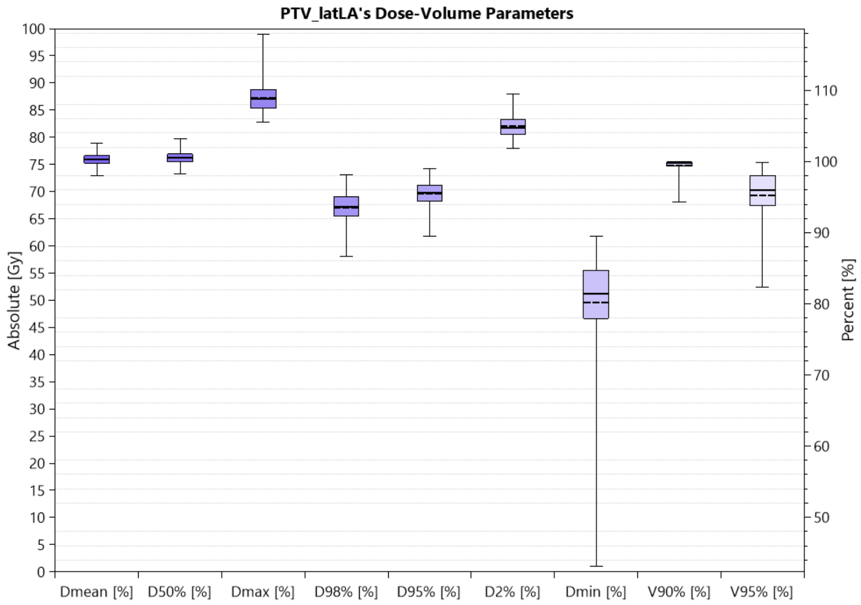

(b) Dose-Volume parameter of PTV_{Supra}

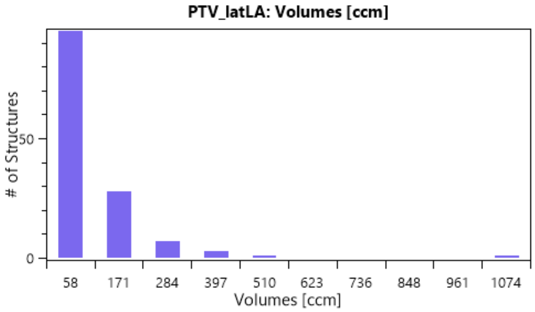

(c) Volumes of PTV_{Supra}

Fig. A4: (a) Median DVH of all matched structures PTV_{Supra} is shown. Additionally specified is the maximum, upper quartile (75%), lower quartile (25%) and minimum of all single DVHs. In (b) Boxplots of the dose-volume parameters and (c) histogram of the volume of all matched structures PTV_{Supra} is shown.

A.3.3 Additional information: PTV_{MI}

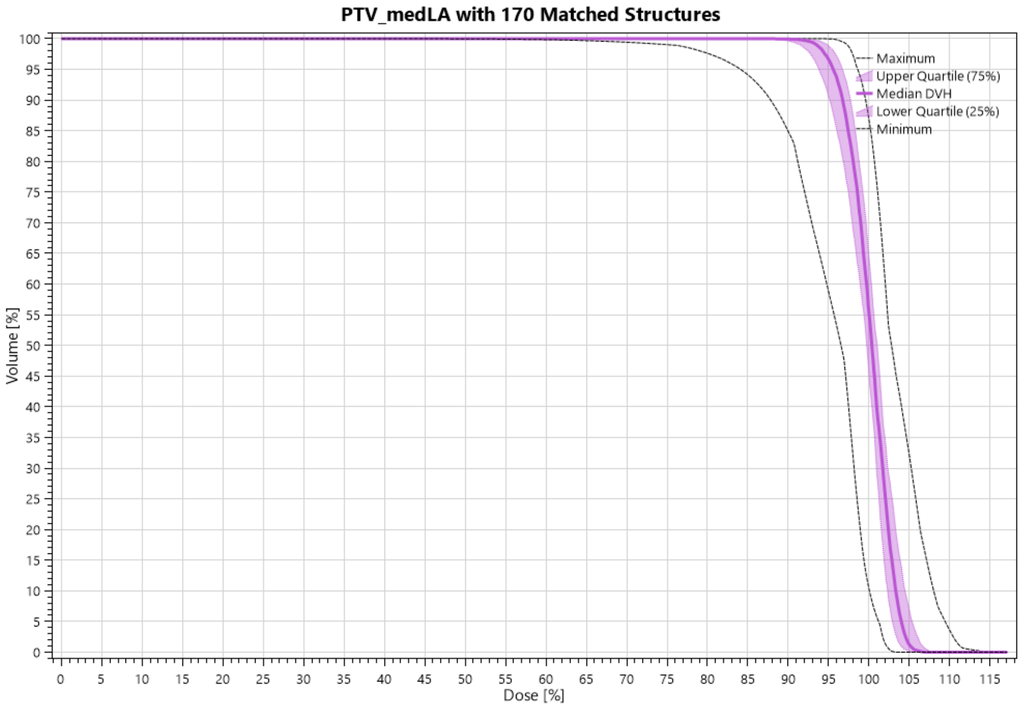

(a) Population DVH of PTV_{MI}

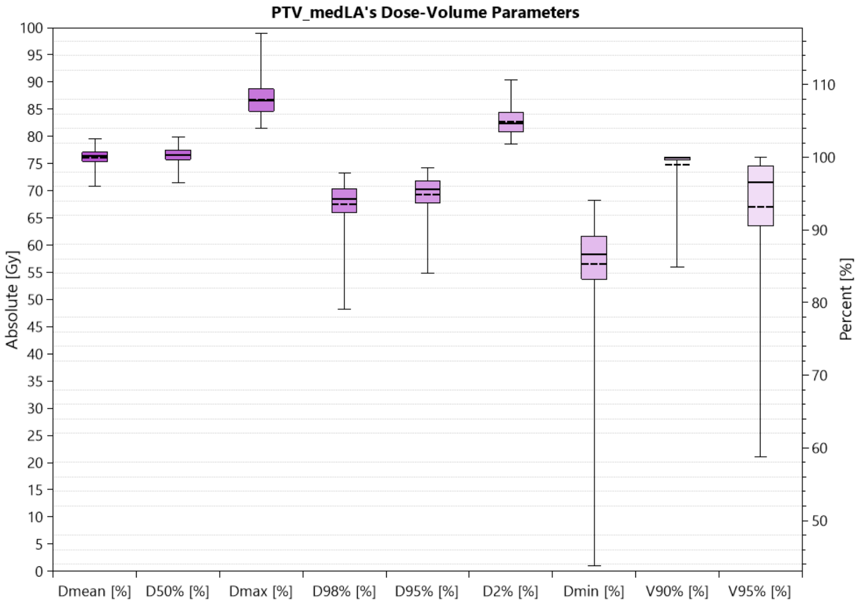

(b) Dose-Volume parameter of PTV_{MI}

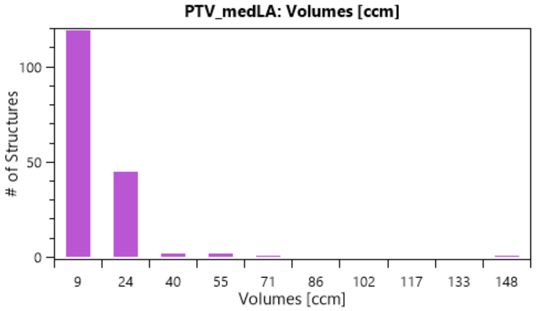

(c) Volumes of PTV_{MI}

Fig. A5: (a) Median DVH of all matched structures PTV_{MI} is shown. Additionally specified is the maximum, upper quartile (75%), lower quartile (25%) and minimum of all single DVHs. In (b) Boxplots of the dose-volume parameters and (c) histogram of the volume of all matched structures PTV_{MI} is shown.

A.3.4 Additional information: Lung total

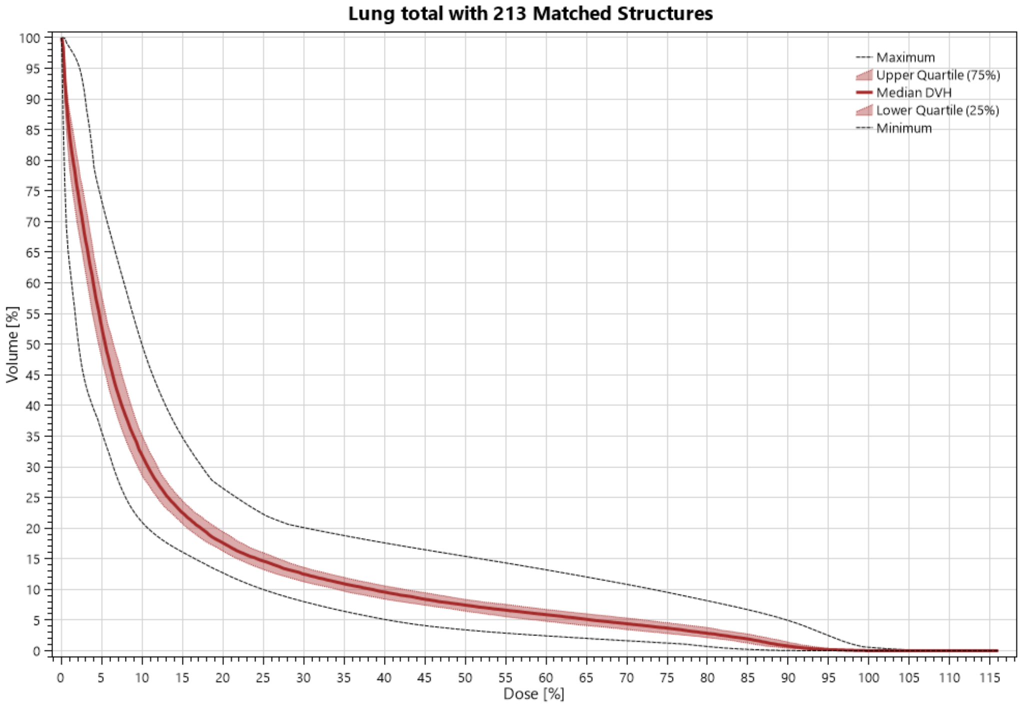

(a) Population DVH

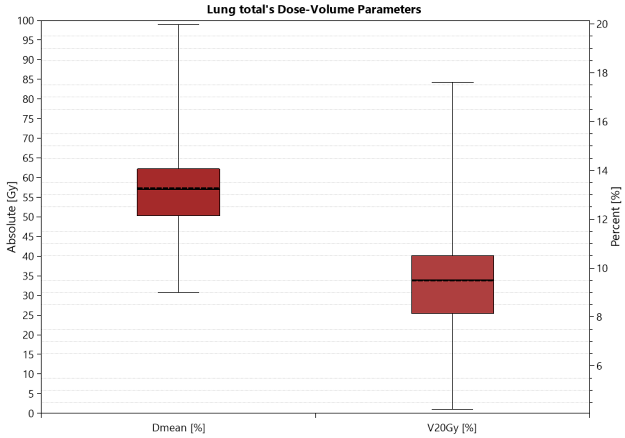

(b) Dose-Volume parameters

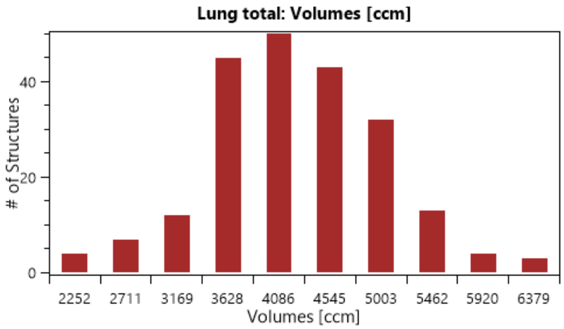

(c) Volume

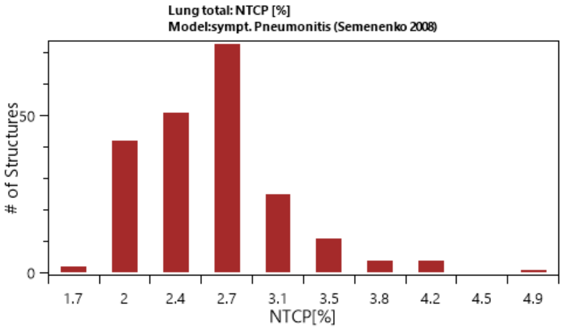

(d) NTCP

Fig. A6: (a) Median DVH of all matched structures *Lung total* is shown. Additionally specified is the maximum, upper quartile (75%), lower quartile (25%) and minimum of all single DVHs. In (b) Boxplots of the dose-volume parameters (c) histogram of the volume and (d) histogram of the NTCP of all matched structures *Lung total* is shown.

A.3.5 Additional information: ipsilateral Lung

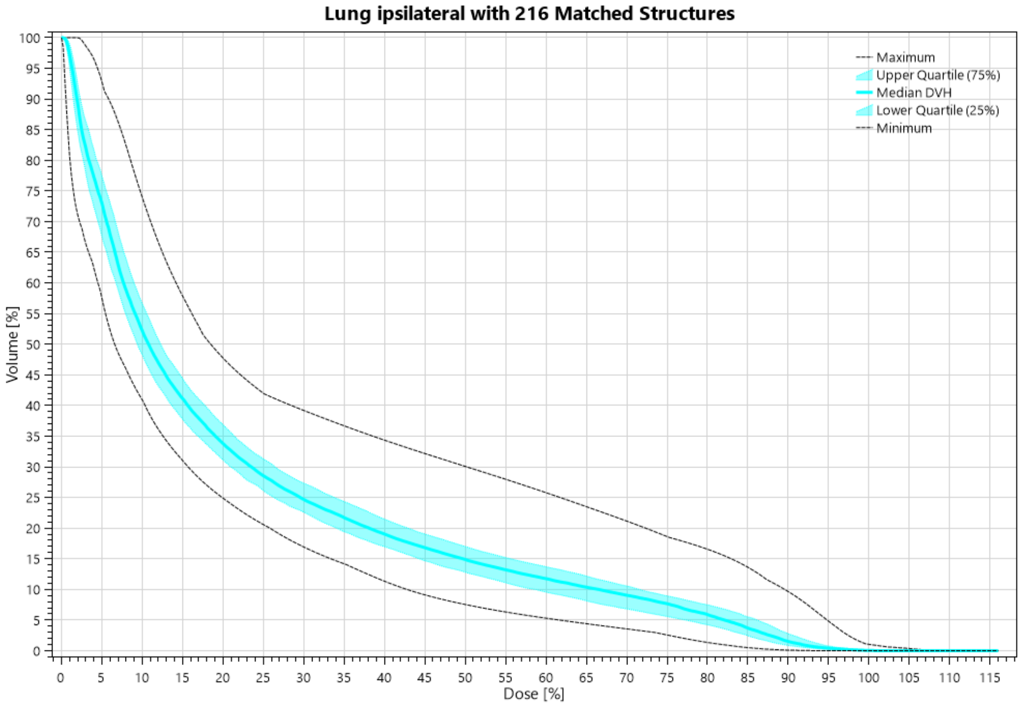

(a) Population DVH

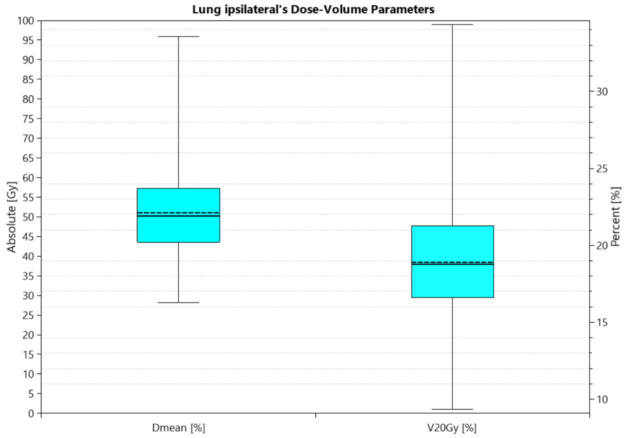

(b) Dose-Volume parameter

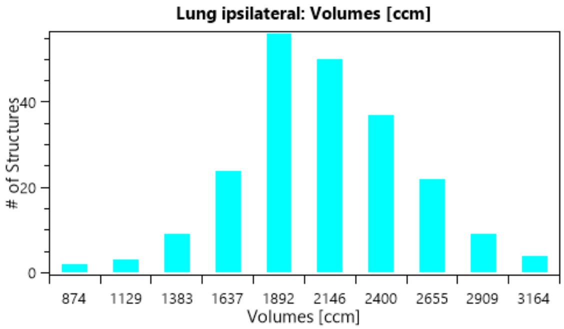

(c) Volume

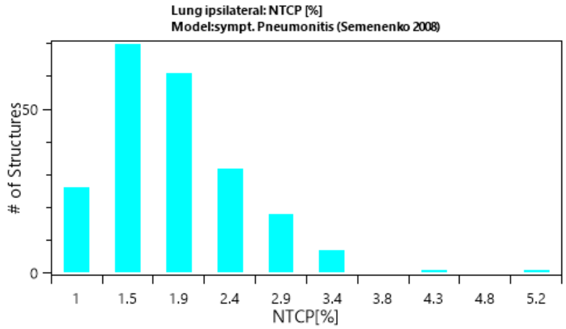

(d) NTCP

Fig. A7: (a) Median DVH of all matched structures *Lung ipsilateral* is shown. Additionally specified is the maximum, upper quartile (75%), lower quartile (25%) and minimum of all single DVHs. In (b) Boxplots of the dose-volume parameters (c) histogram of the volume and (d) histogram of the NTCP of all matched structures *Lung ipsilateral* is shown.

A.3.6 Additional information: contralateral Lung

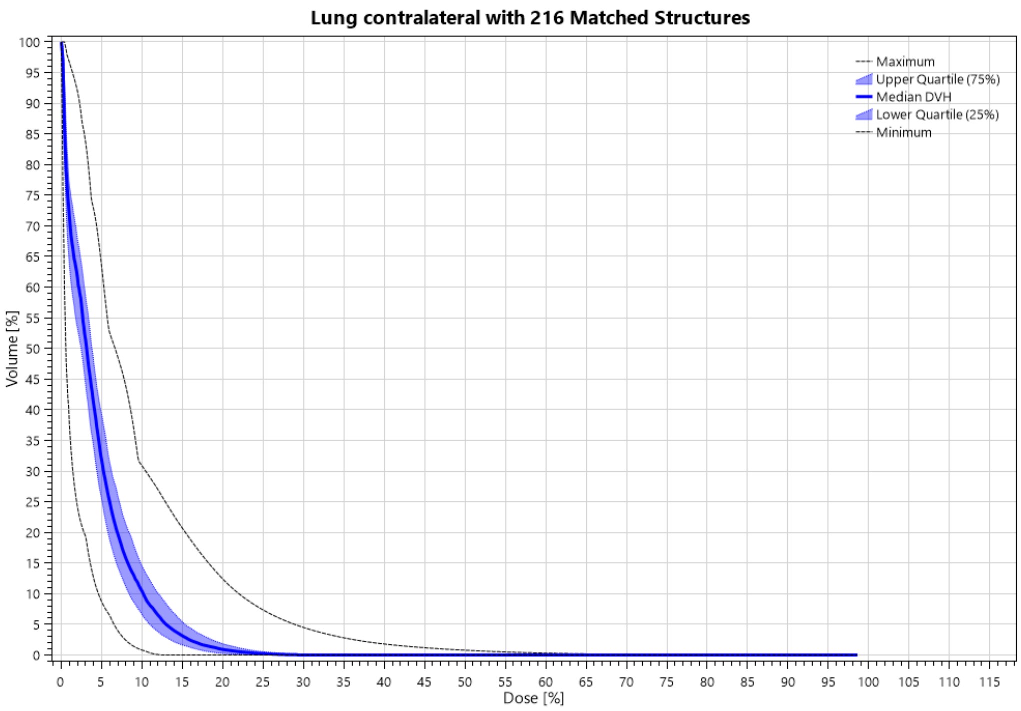

(a) Population DVH

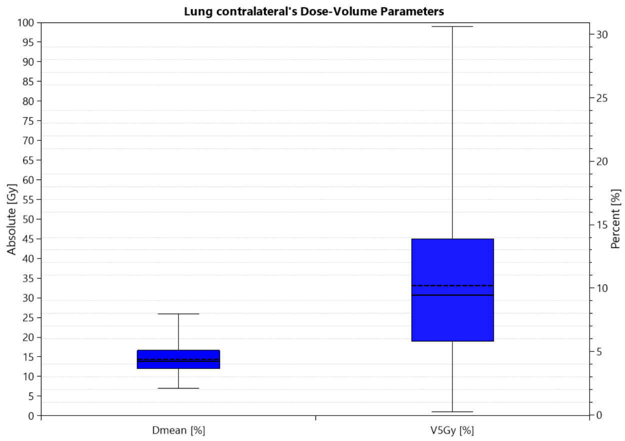

(b) Dose-Volume parameter

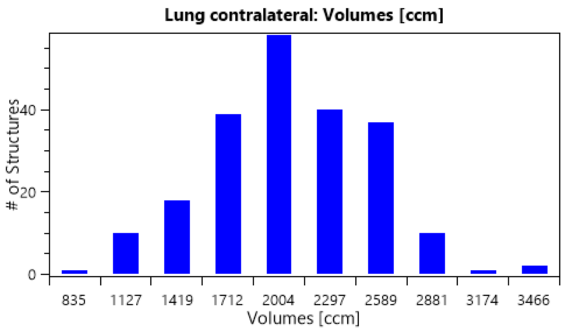

(c) Volume

Fig. A8: (a) Median DVH of all matched structures *Lung contralateral* is shown. Additionally specified is the maximum, upper quartile (75%), lower quartile (25%) and minimum of all single DVHs. In (b) Boxplots of the dose-volume parameters and (c) histogram of the volume of all matched structures *Lung contralateral* is shown.

A.3.7 Additional information: Esophagus

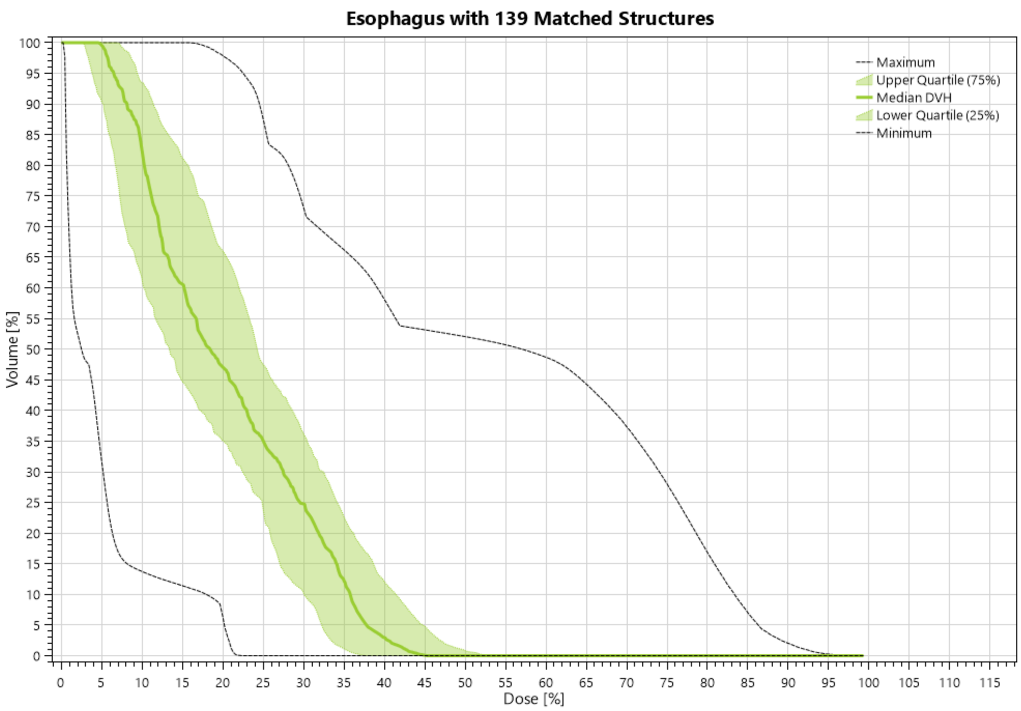

(a) Population DVH

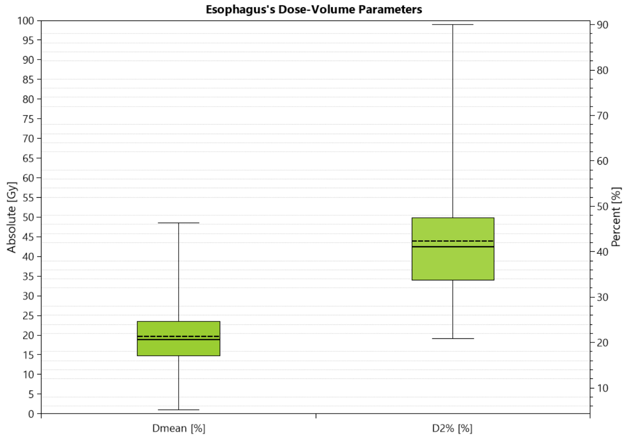

(b) Dose-Volume parameter

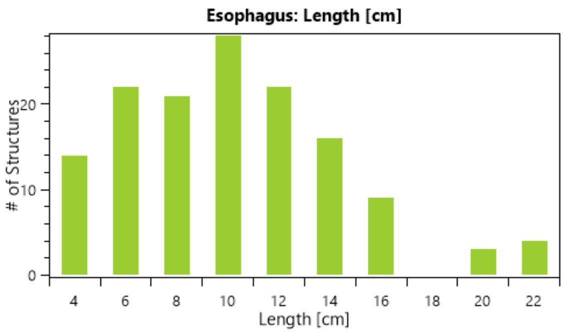

(c) Length

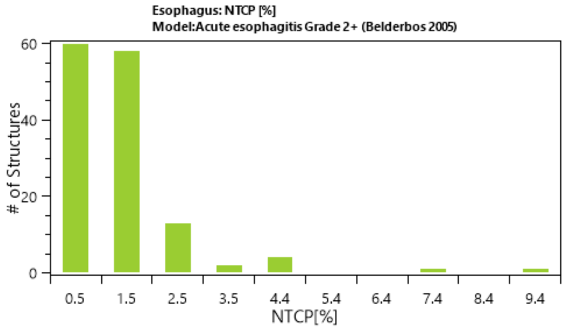

(d) NTCP

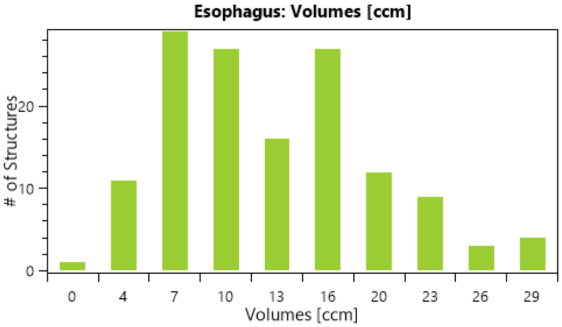

(e) Volume

Fig. A9: (a) Median DVH of all matched structures *Esophagus* is shown. Additionally specified is the maximum, upper quartile (75%), lower quartile (25%) and minimum of all single DVHs. In (b) Boxplots of the dose-volume parameters (c) histogram of the volume, (d) NTCP and (e) histogram of the length of all matched structures *Esophagus* is shown.

A.3.8 Additional information: Heart

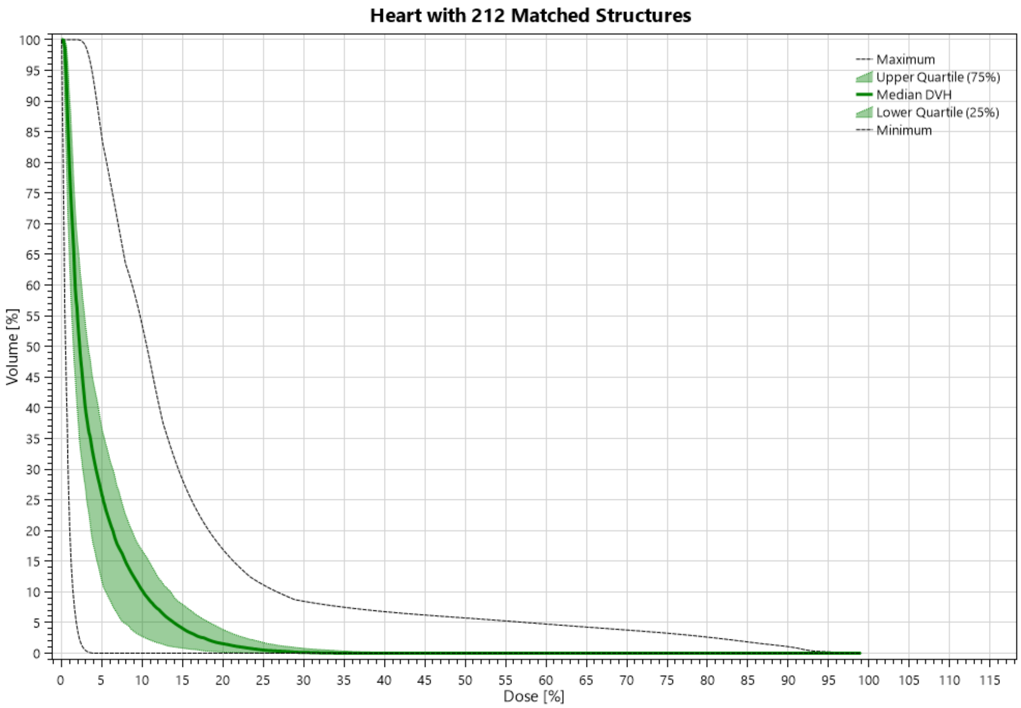

(a) Population DVH

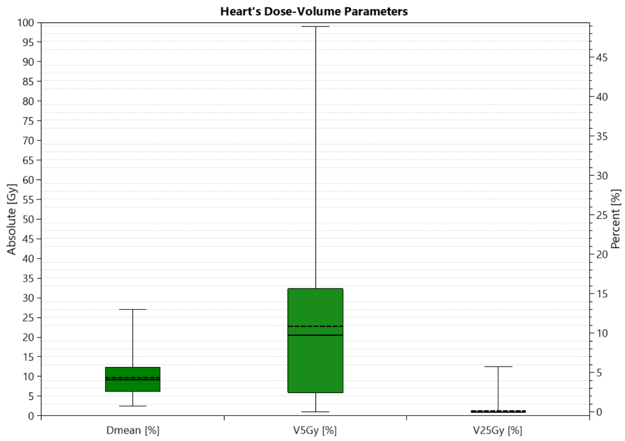

(b) Dose-Volume parameter

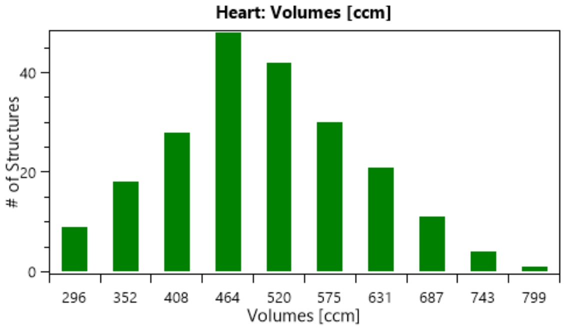

(c) Volume

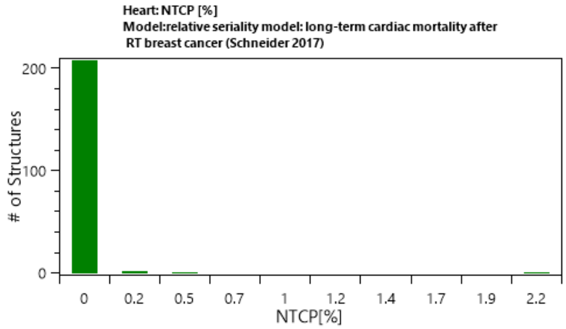

(d) NTCP

Fig. A10: (a) Median DVH of all matched structures *Heart* is shown. Additionally specified is the maximum, upper quartile (75%), lower quartile (25%) and minimum of all single DVHs. In (b) Boxplots of the dose-volume parameters (c) histogram of the volume and (d) histogram of the NTCP of all matched structures *Heart* is shown.

A.3.9 Additional information: Contralateral breast

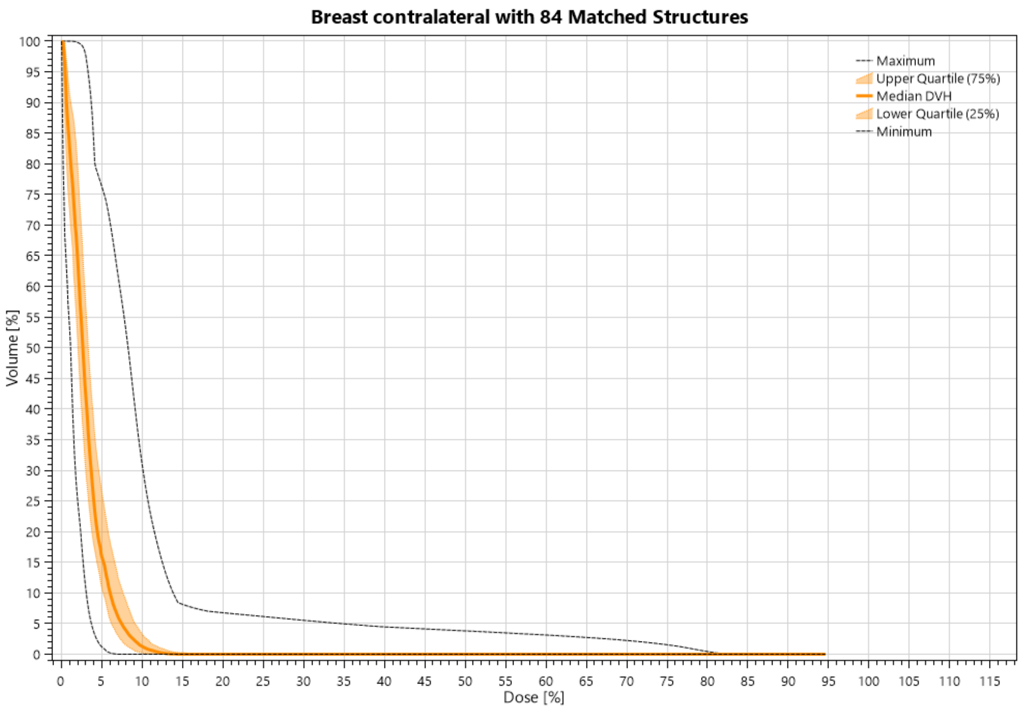

(a) Population DVH

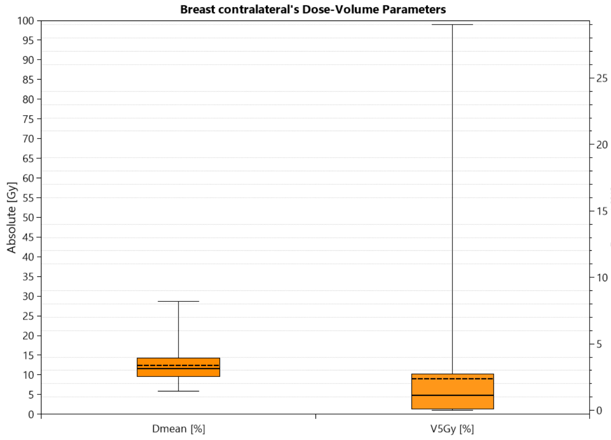

(b) Dose-Volume parameter

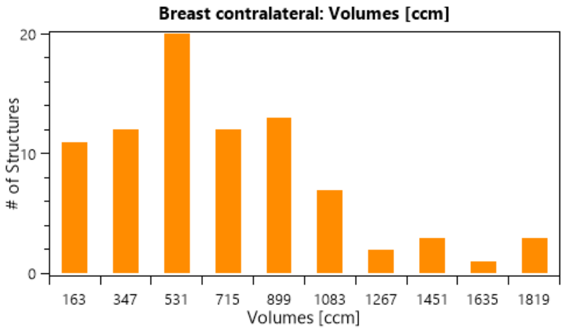

(c) Volume

Fig. A11: (a) Median DVH of all matched structures *Breast contralateral* is shown. Additionally specified is the maximum, upper quartile (75%), lower quartile (25%) and minimum of all single DVHs. In (b) Boxplots of the dose-volume parameters and (c) histogram of the volume of all matched structures *Breast contralateral* is shown.

A.3.10 Additional information: Spinal Cord

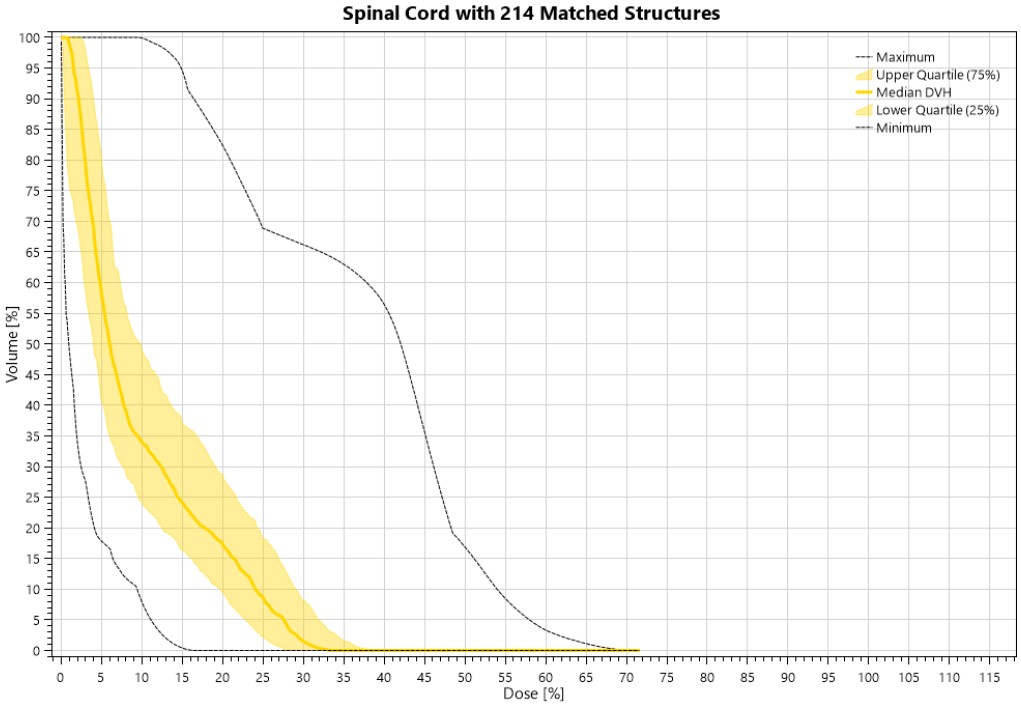

(a) Population DVH

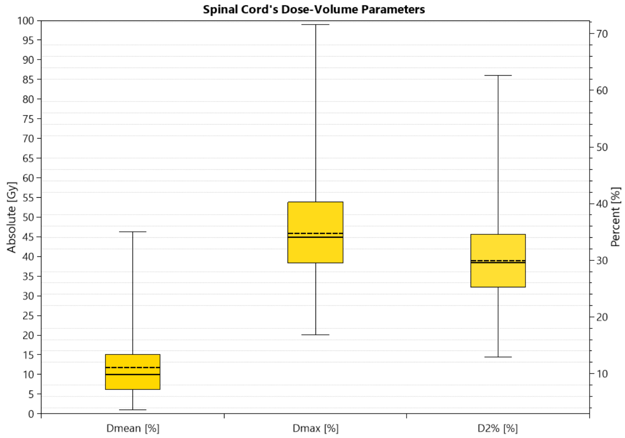

(b) Dose-Volume parameter

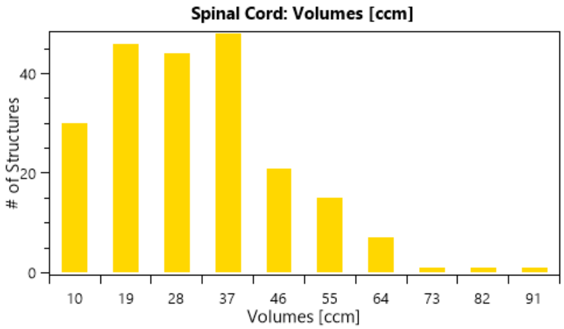

(c) Volume

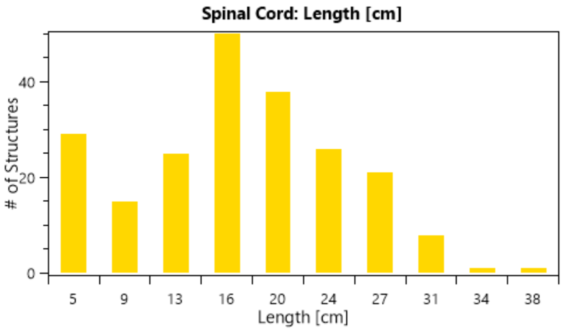

(d) Length

Fig. A12: (a) Median DVH of all matched structures *Spinal Cord* is shown. Additionally specified is the maximum, upper quartile (75%), lower quartile (25%) and minimum of all single DVHs. In (b) Boxplots of the dose-volume parameters (c) histogram of the volume and (d) histogram of the length of all matched structures *Spinal Cord* is shown.

A.4 Mean organ stray doses for the four evaluated planning techniques: hybrid, pure IMRT, VMAT and 3DCRT

		mean organdose [mGy]			
organ	volume [cm^3	hybrid	IMRT	VMAT	3DCRT
Bladder	140.6	43	61	17	28
Small Bowel	778.8	143	182	54	78
Brain	1233.5	164	409	144	155
Colon	364.2	124	166	47	69
Cranium	570.8	177	436	140	171
Ear (Eternals)	5	181	548	152	209
Eyes	13.2	211	487	162	202
Femur lower	272.5	28	56	21	32
Femur upper	210.4	38	56	16	27
Gallbladder	38.3	515	580	225	216
Adrenal Gland	8.1	472	493	205	188
Sublingual Gland	6.7	394	480	669	552
Submandibular Gland	17.8	376	549	707	601
Kidneys	192.3	272	320	113	120
Lenses	0.3	212	494	159	204
Mandible	41	361	567	550	504
Nose	13.4	204	566	103	246
Hip bone	436.7	56	79	23	38
Ovaries	6.7	50	69	18	32
Pancreas	81.1	374	430	169	150
Parotids	37.2	297	459	180	323
Pituitary Gland	0.4	196	447	168	189
Sacrum	134.4	69	95	30	51
Spleen	85.1	529	535	136	261
Stomach	251.6	516	561	189	228
Tibiae fibiae	356.6	12	21	8	11
Tongue	54.7	446	679	649	658
Tonsil	2.4	304	692	197	332
Uterus	52.3	45	64	16	30

Tab. A1: The Comparison of mean organ doses [mGy] for a complete treatment with 50Gy prescribed dose in the stray dose regime.
